# The whole-genome landscape of medulloblastoma subtypes

**DOI:** 10.1038/nature22973

**Published:** 2017-07-20

**Authors:** Paul A. Northcott, Ivo Buchhalter, A. Sorana Morrissy, Volker Hovestadt, Joachim Weischenfeldt, Tobias Ehrenberger, Susanne Gröbner, Maia Segura-Wang, Thomas Zichner, Vasilisa A. Rudneva, Hans-Jörg Warnatz, Nikos Sidiropoulos, Aaron H. Phillips, Steven Schumacher, Kortine Kleinheinz, Sebastian M. Waszak, Serap Erkek, David T. W. Jones, Barbara C. Worst, Marcel Kool, Marc Zapatka, Natalie Jäger, Lukas Chavez, Barbara Hutter, Matthias Bieg, Nagarajan Paramasivam, Michael Heinold, Zuguang Gu, Naveed Ishaque, Christina Jäger-Schmidt, Charles D. Imbusch, Alke Jugold, Daniel Hübschmann, Thomas Risch, Vyacheslav Amstislavskiy, Francisco German Rodriguez Gonzalez, Ursula D. Weber, Stephan Wolf, Giles W. Robinson, Xin Zhou, Gang Wu, David Finkelstein, Yanling Liu, Florence M. G. Cavalli, Betty Luu, Vijay Ramaswamy, Xiaochong Wu, Jan Koster, Marina Ryzhova, Yoon-Jae Cho, Scott L. Pomeroy, Christel Herold-Mende, Martin Schuhmann, Martin Ebinger, Linda M. Liau, Jaume Mora, Roger E. McLendon, Nada Jabado, Toshihiro Kumabe, Eric Chuah, Yussanne Ma, Richard A. Moore, Andrew J. Mungall, Karen L. Mungall, Nina Thiessen, Kane Tse, Tina Wong, Steven J. M. Jones, Olaf Witt, Till Milde, Andreas Von Deimling, David Capper, Andrey Korshunov, Marie-Laure Yaspo, Richard Kriwacki, Amar Gajjar, Jinghui Zhang, Rameen Beroukhim, Ernest Fraenkel, Jan O. Korbel, Benedikt Brors, Matthias Schlesner, Roland Eils, Marco A. Marra, Stefan M. Pfister, Michael D. Taylor, Peter Lichter

**Affiliations:** 1 Division of Pediatric Neurooncology, German Cancer Research Center (DKFZ), Heidelberg, Germany; 2 Department of Developmental Neurobiology, St Jude Children’s Research Hospital, Memphis, Tennessee USA; 3 Division of Theoretical Bioinformatics, German Cancer Research Center (DKFZ), Heidelberg, Germany; 4 Division of Applied Bioinformatics, German Cancer Research Center (DKFZ), Heidelberg, Germany; 5 Department for Bioinformatics and Functional Genomics, Institute for Pharmacy and Molecular Biotechnology (IPMB) and BioQuant, Heidelberg University, Heidelberg, Germany; 6 Developmental & Stem Cell Biology Program, The Hospital for Sick Children, Toronto, Ontario; 7 Division of Molecular Genetics, German Cancer Research Center (DKFZ), Heidelberg, Germany; 8 Biotech Research & Innovation Centre (BRIC), Copenhagen University and Finsen Laboratory, Rigshospitalet, Denmark; 9 Department of Biological Engineering, Massachusetts Institute of Technology, Cambridge, Massachusetts USA; 10 German Cancer Consortium (DKTK), Heidelberg, Germany; 11 Genome Biology Unit, European Molecular Biology Laboratory (EMBL), Heidelberg, Germany; 12 Department of Vertebrate Genomics, Max Planck Institute for Molecular Genetics, Berlin, Germany; 13 Department of Structural Biology, St Jude Children’s Research Hospital, Memphis, Tennessee USA; 14 Broad Institute of Harvard and MIT, Cambridge, Massachusetts USA; 15 Heidelberg Center for Personalized Oncology (DKFZ-HIPO), German Cancer Research Center (DKFZ), Heidelberg, Germany; 16 Medical Faculty Heidelberg, Heidelberg University, Heidelberg, Germany; 17 Department of Pediatric Hematology and Oncology, Heidelberg University Hospital, Heidelberg, Germany; 18 Genomics and Proteomics Core Facility, German Cancer Research Center (DKFZ), Heidelberg, Germany; 19 Department of Oncology, St Jude Children’s Research Hospital, Memphis, Tennessee USA; 20 Department of Computational Biology, St Jude Children’s Research Hospital, Memphis, Tennessee USA; 21 Department of Oncogenomics, Amsterdam Medical Center, Amsterdam, Netherlands; 22 Department of Neuropathology, NN Burdenko Neurosurgical Institute, Moscow, Russia; 23 Department of Pediatrics, Papé Family Pediatric Research Institute, Knight Cancer Institute, Oregon Health and Science University, Portland, Oregon USA; 24 Department of Neurology, Boston Children's Hospital and Harvard Medical School, Boston Massachusetts USA; 25 Department of Neurosurgery, University Clinic, Heidelberg University, Heidelberg Hospital, Germany; 26 Department of Neurosurgery, University Hospital Tübingen, Tübingen, Germany; 27 Department of Hematology and Oncology, Children’s University Hospital Tübingen, Tübingen, Germany; 28 Department of Neurosurgery, David Geffen School of Medicine at UCLA, Los Angeles, California USA; 29 Developmental Tumor Biology Laboratory, Hospital Sant Joan de Déu, Barcelona, Spain; 30 Department of Pathology, Duke University, Durham, North County USA; 31 Department of Pediatrics, McGill University, Montreal, Quebec Canada; 32 Department of Neurosurgery, Kitasato University School of Medicine, Sagamihara, Japan; 33 Michael Smith Genome Sciences Centre, BC Cancer Agency, Vancouver, British Columbia Canada; 34 Department of Neuropathology, Heidelberg University Hospital, Heidelberg, Germany; 35 Division of Neurosurgery, Hospital for Sick Children, Toronto, Ontario Canada

**Keywords:** Cancer genomics, CNS cancer

## Abstract

Current therapies for medulloblastoma, a highly malignant childhood brain tumour, impose debilitating effects on the developing child, and highlight the need for molecularly targeted treatments with reduced toxicity. Previous studies have been unable to identify the full spectrum of driver genes and molecular processes that operate in medulloblastoma subgroups. Here we analyse the somatic landscape across 491 sequenced medulloblastoma samples and the molecular heterogeneity among 1,256 epigenetically analysed cases, and identify subgroup-specific driver alterations that include previously undiscovered actionable targets. Driver mutations were confidently assigned to most patients belonging to Group 3 and Group 4 medulloblastoma subgroups, greatly enhancing previous knowledge. New molecular subtypes were differentially enriched for specific driver events, including hotspot in-frame insertions that target *KBTBD4* and ‘enhancer hijacking’ events that activate *PRDM6*. Thus, the application of integrative genomics to an extensive cohort of clinical samples derived from a single childhood cancer entity revealed a series of cancer genes and biologically relevant subtype diversity that represent attractive therapeutic targets for the treatment of patients with medulloblastoma.

**Supplementary information:**

The online version of this article (doi:10.1038/nature22973) contains supplementary material, which is available to authorized users.

## Abstract (web summary)

Genomic analysis of 491 medulloblastoma samples, including methylation profiling of 1,256 cases, effectively assigns candidate drivers to most tumours across all molecular subgroups.

**Supplementary information:**

The online version of this article (doi:10.1038/nature22973) contains supplementary material, which is available to authorized users.

## Genomic landscapes of medulloblastomas

Medulloblastomas are highly malignant brain tumours that develop during childhood. Paul Northcott and colleagues analysed the whole-genome sequences of 491 medulloblastomas in order to characterize the genomic landscape across tumours and identify new drivers and mutational signatures. Their integrative genomic analyses, including methylation profiling of 1,256 medulloblastomas, identifies subgroup-specific driver mutations and suggests additional tumour subtypes. The authors assign driver mutations to a high proportion of the less well characterized Group 3 and Group 4, which together contribute to more than 60% of all medulloblastomas.

**Supplementary information:**

The online version of this article (doi:10.1038/nature22973) contains supplementary material, which is available to authorized users.

## Main

Next-generation sequencing (NGS) studies have tremendously advanced our understanding of the genes, pathways and molecular processes that underly most commonly diagnosed human cancers. These efforts have identified core sets of ‘driver’ genes that are frequently mutated across a wide spectrum of different cancer entities^1,2^. Although the genetic underpinnings of some cancers were largely resolved during the first ‘wave’ of NGS studies, especially for comparatively simple malignancies driven by deregulation of a single pathway^3,4^, others remain enigmatic and require further interrogation with sufficient power to overcome confounding molecular heterogeneity and diversity.

Medulloblastoma (MB) (World Health Organization grade IV) is a highly malignant childhood brain tumour that has been the subject of several NGS studies conducted by the International Cancer Genome Consortium (ICGC)^5,6,7,8^, the Paediatric Cancer Genome Project (PCGP)^9^, and the Medulloblastoma Advanced Genomics Consortium (MAGIC)^10,11^. Consensus molecular subgroups of MB, namely WNT, SHH, Group 3 and Group 4, exhibit distinctive transcriptional and epigenetic signatures that define clinically relevant patient subsets^12,13^. WNT and SHH subgroup MBs are primarily driven by mutations leading to constitutive activation of the Wingless and Sonic hedgehog signalling pathways, respectively. By contrast, the genetics and biology underlying the Group 3 and Group 4 MB subgroups remain less clear^12^. Targeted therapies for MB are scarce yet desperately needed, warranting intensive investigation into the full range of genetic lesions and molecular heterogeneity that contribute to MB subgroups, especially as it relates to poorly characterized Group 3 and Group 4 disease. Here we report the genomic landscape across a series of 491 previously untreated MBs. Our comprehensive and integrative approach to this multilayered dataset provides considerable biological insight into each of the core subgroups, including the identification of new subgroup-specific driver genes, epigenetic subtypes, and candidate targets for therapy. This dataset provides a rich resource for the cancer genomics community and will serve as the foundation of ongoing and future candidate-driven functional studies focused on resolving MB aetiology.

## Patient cohorts and genomic datasets

**Figure 1:**
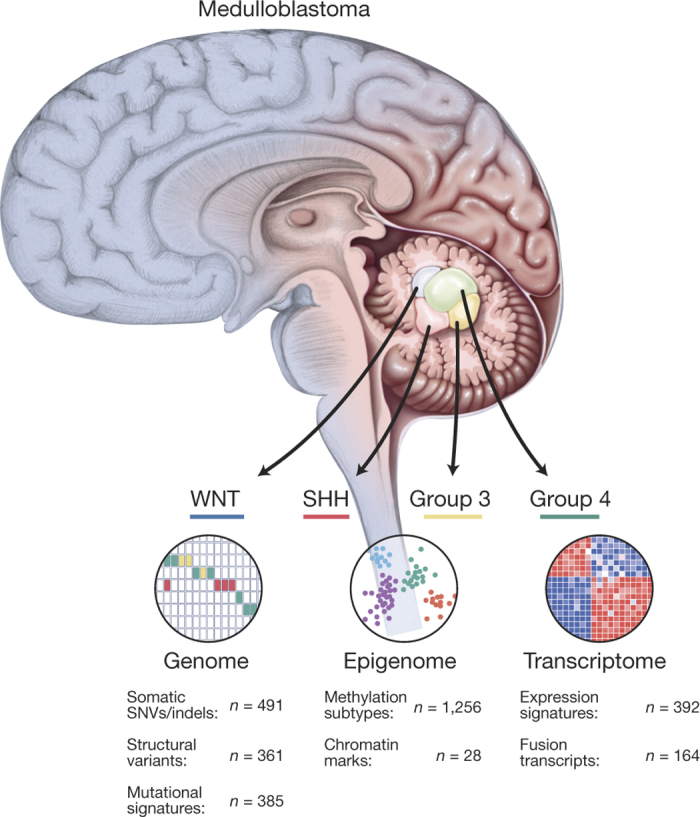
Summary of MB genomic datasets. Graphical summary of genomic, epigenomic, and transcriptomic MB datasets analysed in the study. PowerPoint slide

Patient-matched tumour and non-tumour (blood) Illumina DNA sequences were collected from a total of 579 untreated patients diagnosed with MB that were sequenced at one of four participating institutions (see Methods). After eliminating samples with poor quality sequencing data (based on quality control measures), samples sequenced more than once (that is, duplicate cases analysed at different sequencing sites or sequencing of patient-matched primary and relapse pairs), samples lacking molecular subgroup annotation, and cases with clear molecular evidence for misdiagnosis, we amassed a final cohort of 491 diagnostic MBs with matched normal samples for further analysis, including standardized sequence alignment and filtering, as well as harmonized single nucleotide variant (SNV), indel, and structural variant calling (Fig. 1). Germline and somatic alterations were annotated primarily from whole genomes (*n* = 390; *n* = 190 published^5,6,9^ and 200 unpublished) while the remaining alterations were derived from published whole exomes (*n* = 101)^5,14^. Patient ages ranged from 1 month to 50 years (median age = 8 years; Supplementary Table 1). Verification of MB diagnosis and subgroup status was established using a molecular classification approach based on DNA methylation arrays^15^ (see Methods). Illumina 450 k methylation array data were generated for 1,256 MBs, including 396 out of 491 (80.7%) of the NGS cohort. Transcriptome data were acquired through RNA sequencing (RNA-seq; *n* = 164) and Affymetrix expression arrays (*n* = 392). Chromatin immunoprecipitation followed by sequencing (ChIP–seq) data were generated for several chromatin marks (such as H3K27ac and CTCF) on a subset of the cohort (Supplementary Table 1). Mutations, structural variants and supporting epigenetic and transcriptional data can be freely explored online through multiple data portals (see ‘Data availability’ in Methods).

## Mutational signatures operative in MB

**Extended Data Figure 1:**
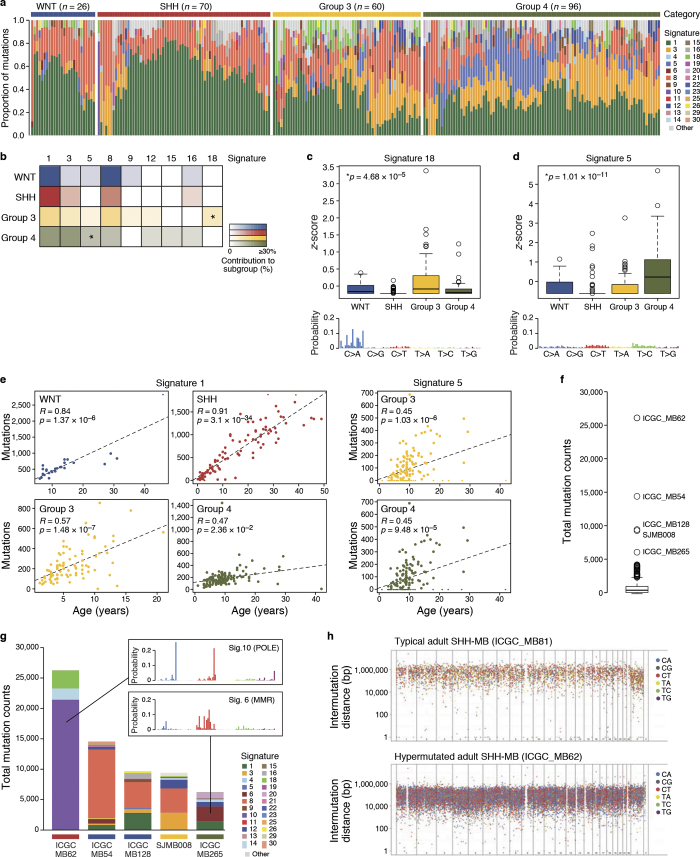
Mutational signatures in MB. **a**, **b**, Exposure plot (**a**) and heatmap (**b**) summarizing mutation signatures contributing ≥5% of the overall mutation burden per sample are depicted. Asterisks in **b** indicate subgroup-enriched signatures. **c**, **d**, Box plots showing the subgroup specificity of signatures 18 (**c**) and 5 (**d**). **e**, Correlation of signatures 1 and 5 with patient age. **f**, Summary of total somatic mutation counts observed in the series. **g**, Bar plot summarizing distribution of mutation signatures in MBs with outlier mutation counts. **h**, Rainfall plots depicting somatic mutation burden in typical (top) and hypermutated (bottom) SHH MBs.

Mutational signatures have been extensively catalogued across a broad spectrum of cancerous tissues, and for many of these, underlying exogenous and endogenous processes have been described. A seminal pan-cancer analysis that included 100 MBs (not split by subgroup) revealed three predominant signatures active in MB^16^: signatures 1, 5 and 8. Here we analysed a total of 440,459 somatic mutations across 385 MB genomes divided into molecular subgroups (5 out of 390 whole-genome sequencing (WGS) cases were excluded owing to quality control issues) and detected 24 signatures with a mutation contribution of at least 5% in one or more samples (Extended Data Fig. 1a). Signature 1, associated with patient age at diagnosis, was the most common signature in all subgroups (Extended Data Fig. 1a, b). Signature 3, which was not previously detected in MB^16^ and has been linked with underlying *BRCA1* and *BRCA2* mutations in breast, aggressive prostate, and pancreatic cancers^16,17^, was unexpectedly observed in most of the patients with Group 3 and Group 4 MB, and a subset of patients with SHH MB. Additional subgroup-enriched signatures included signatures 18 (Group 3; *P* = 4.7 × 10^−5^) and 5 (Group 4; *P* = 1.0 × 10^−11^), the latter being positively correlated with patient age (Extended Data Fig. 1a–e).

Five MBs were identified as potential outliers with respect to mutation burden, harbouring more than 5,000 somatic mutations per genome compared to the median of 698 mutations per genome observed for the entire cohort (Extended Data Fig. 1f, g). ICGC_MB62 (SHH) harboured over 25,000 mutations and was the only MB we considered a bona fide hypermutator among the cohort (Extended Data Fig. 1f–h). Most somatic mutations in this patient were C>A and C>T substitutions, known to be distinct peaks in signature 10 and consistent with altered activity of the replication factor DNA polymerase ε, encoded by *POLE*, which we determined to be somatically mutated^18,19^. Similarly, ICGC_MB265 exhibited a disproportionally high mutation load ascribed to signature 6 (Extended Data Fig. 1f, g), the latter reported to be associated with mismatch repair deficiencies and explained by the somatic *MLH1* mutation we identified in this patient.

## Subgroup-specific drivers and pathways

**Figure 2:**
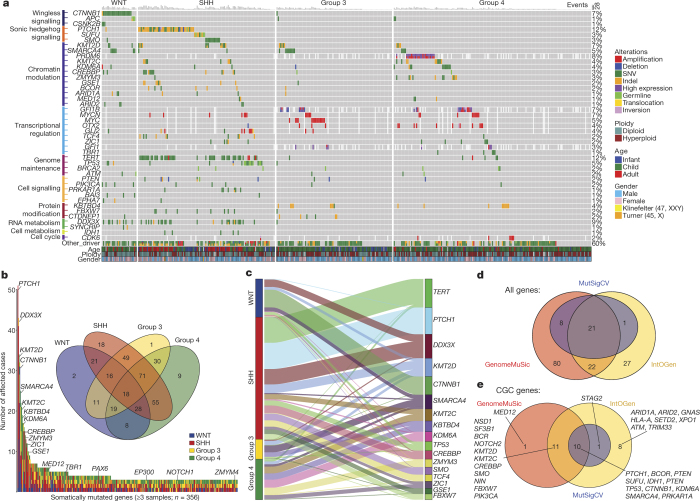
Driver genes and pathways altered in MB. **a**, Oncoprint summarizing recurrently altered genes according to MB subgroup (*n* = 390; WGS series only). **b**, Top, Venn diagram summarizing the subgroup overlap of recurrently mutated genes (≥3 affected cases). Bottom, incidence plot of recurrently mutated genes (≥3 affected cases) detected in the series (*n* = 356 genes; *n* = 491 samples). **c**, Graphical summary of the most frequently mutated genes (≥10 affected cases) and their subgroup distribution. **d**, Venn diagram summarizing the significantly mutated gene lists output from multiple significance algorithms. **e**, Results from **d** restricted to Cancer Gene Census (CGC) genes. PowerPoint slide

**Extended Data Figure 2:**
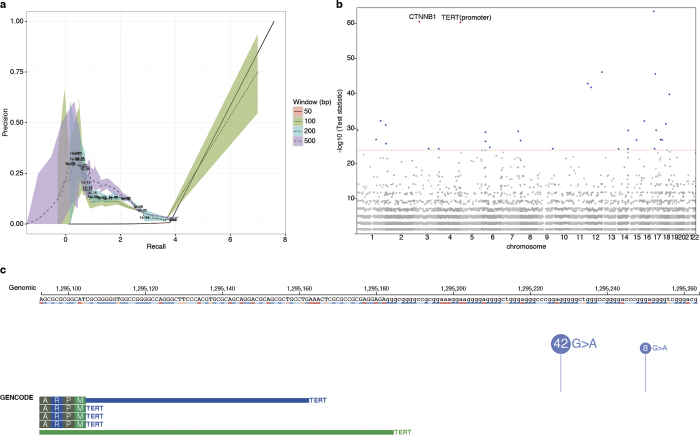
Genome-wide summary of somatic SNVs. **a**, Precision-recall curves for different binomial *P* value cut-offs. Minimal and maximal precision values are shown in colour, mean precision is shown as dotted line. *P* value cut-offs for 200 bp window sizes are indicated. **b**, Manhattan plot showing the −log_10_ test statistic of 200 bp genomic windows plotted against their respective chromosomal positions. Red line indicates the genome-wide significance threshold (*P* = 10^−25^). High-confidence regions are shown in red; regions representing probable false positives are shown in blue. **c**, Summary of *TERT* promoter mutations observed in the series.

**Extended Data Figure 3:**
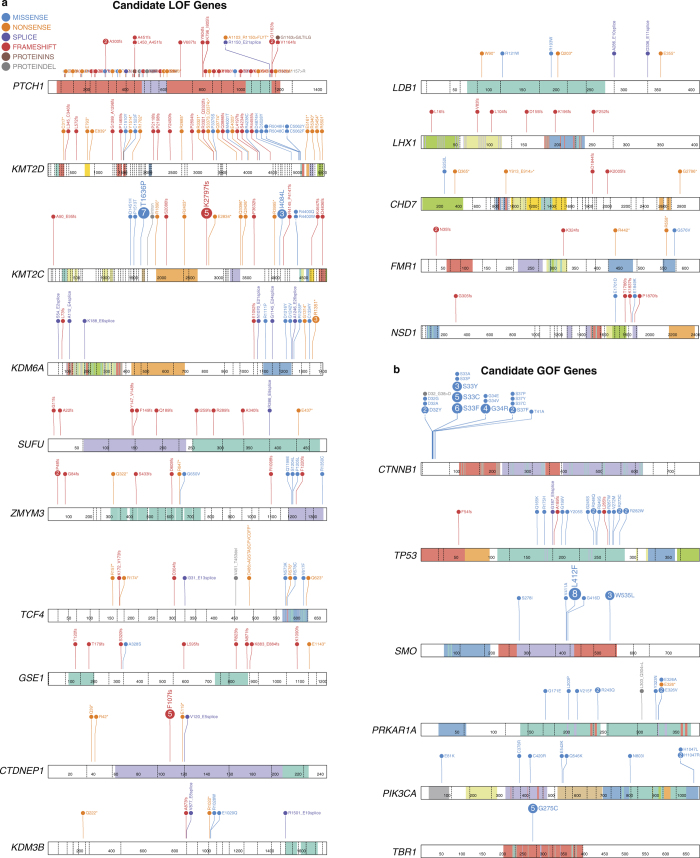
Prevalent candidate driver mutations observed in MB. **a**, **b**, Gene-level summaries of SNVs/indels inferred to predominantly result in loss-of-function (LOF) (**a**) or gain-of-function (GOF) (**b**) of known and putative MB driver genes.

**Extended Data Figure 4:**
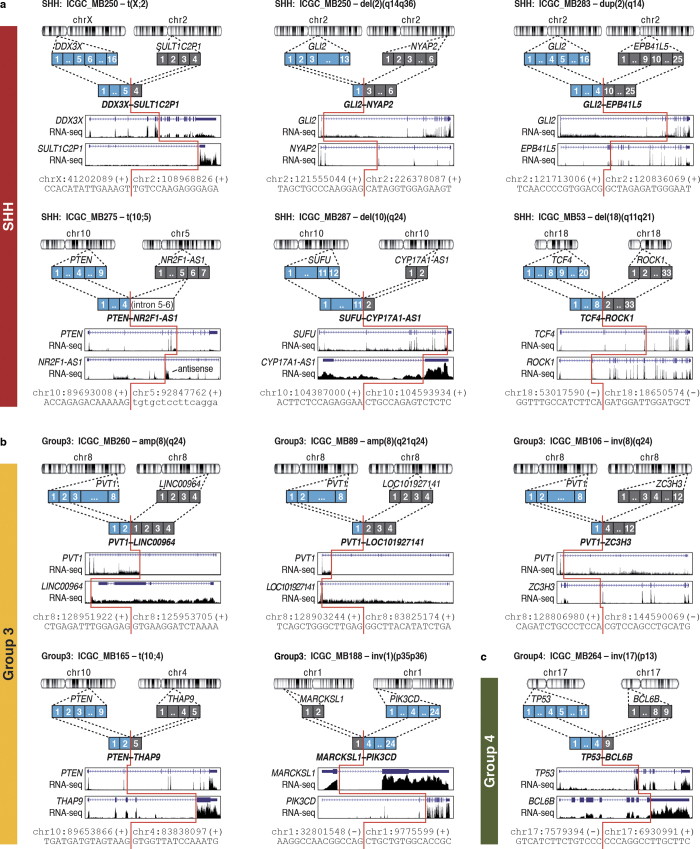
Summary of fusion gene transcripts detected by RNA-seq. **a**–**c**, Schematic summaries of high-confidence fusion transcripts targeting known or putative MB driver genes, organized according to MB subgroup.

Genome-wide analysis of somatic SNVs identified known hotspot variants in the *TERT* promoter as the only confident region of significant mutation in the noncoding MB genome (Extended Data Fig. 2a–c; Supplementary Table 2). In the coding space and as expected, several known and presumed MB driver genes were revealed in our series, including *PTCH1*, *DDX3X*, *KMT2D* (formerly known as *MLL2*) and others, many of which showed clear subgroup-specificity (Fig. 2a–c; Extended Data Fig. 3a, b; Supplementary Table 2). Most recurrently mutated genes contributed to fewer than ten patients each (≤5%), revealing a long ‘tail’ of low-frequency gene alterations that may have unappreciated yet crucial roles during MB pathogenesis. Structural variants generating fusion transcripts that involve known MB driver genes were detected by integrating patient-matched structural variant breakpoints with transcriptome sequencing data, including rare events that recurrently target *GLI2*, *PTEN* and *PVT1* (Extended Data Fig. 4; Supplementary Table 2). Pathogenic germline variants that affect known MB predisposition genes including *PTCH1*, *SUFU* and *TP53* were predominantly restricted to patients with SHH MB and are extensively detailed in a parallel study investigating more than 1,000 MB germ lines (Supplementary Table 2) (S.M.W. *et al*., manuscript submitted).

**Extended Data Figure 5:**
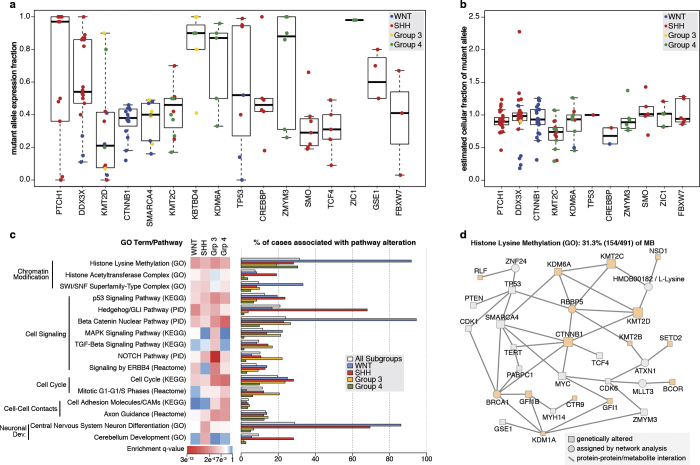
Candidate driver genes and pathways in MB subgroups. **a**, **b**, Box plots summarizing allelic expression fractions (**a**) and estimated clonality (**b**) inferred for prominent MB driver gene mutations. **c**, GO and pathway summary of recurrently mutated genes in MB. GO and pathway categories are grouped according to functional theme and the proportion of cases affected by individual pathway alterations are plotted per subgroup and across the series. **d**, Network summary of recurrently mutated genes involved in histone lysine methylation (GO accession 0034968).

To discriminate potential drivers from passengers, we annotated our dataset using MutSigCV^20^, GenomeMuSiC^21^ and IntOGen^22^ significance algorithms (Fig. 2d; Supplementary Table 2). Overlapping the output derived from these analyses identified a core set of high-confidence somatic drivers detected by all three algorithms (*n* = 21 genes). However, no single significance algorithm demonstrated superior sensitivity compared to the others, and many bona fide cancer genes failed detection by one or more methods, substantiating our approach of unifying results from all three pipelines (Fig. 2e). Most candidate driver gene mutations were expressed, although often exhibiting variable expression of the mutant allele (that is, *PTCH1* and *DDX3X* mutations in patients with SHH MB; Extended Data Fig. 5a). Similarly, most putative driver gene variants appeared to be clonal (that is, estimated cellular fraction of approximately 1.0; see Methods), with some exceptions, most notably *DDX3X* mutations in patients with WNT MB (Extended Data Fig. 5b).

Consistent with our previous studies^23,24^, genetic events targeting histone modifiers, especially those regulating lysine methylation and/or acetylation, were found across subgroups and contributed to a considerable proportion of cases (Extended Data Fig. 5c, d), further corroborating the hypothesis that deregulation of the epigenetic machinery is fundamental to MB development.

## WNT subgroup MB

**Extended Data Figure 6:**
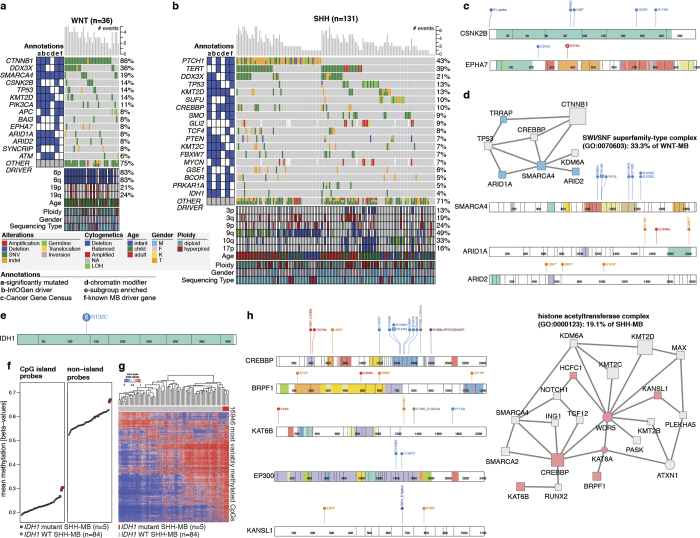
Mutational landscape of WNT and SHH MB. **a**, **b**, Oncoprint summaries of recurrently mutated genes and cytogenetic alterations in WNT (**a**; *n* = 36 samples) and SHH (**b**; *n* = 131 samples). **c**, Gene-level summary of WNT subgroup-enriched *CSNK2B* and *EPHA7* mutations. **d**, Summary of SWI/SNF superfamily-type complex (GO accession 0070603) mutations observed in patients with WNT MB. **e**, Gene-level summary of somatic *IDH1* R132C mutations detected in MB. **f**, Quantification of methylcytosine beta-values detected in *IDH1*-mutant versus wild-type SHH MBs. **g**, Unsupervised hierarchical clustering of SHH MB methylation data, confirming CIMP in *IDH1*-mutated SHH MBs. **h**, Summary of histone acetyltransferase complex (GO accession 0000123) mutations observed in patients with SHH MB.

**Extended Data Figure 7:**
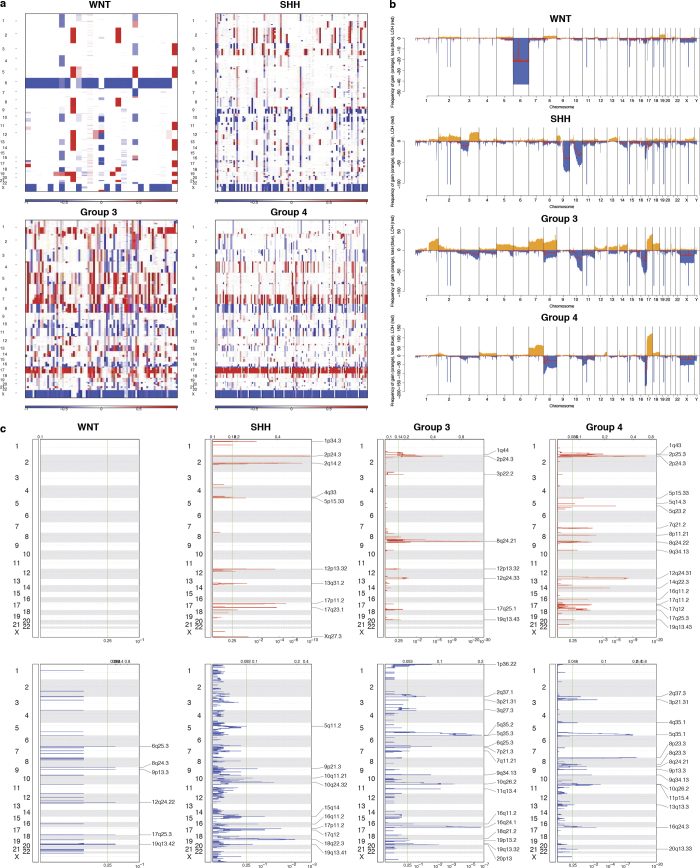
Somatic copy-number alterations in MB. **a**, Copy-number heat maps for individual MB subgroups derived from WGS series. **b**, Genome-wide copy-number summary plots for the MB dataset shown in **a**. **c**, GISTIC plots summarizing significant CNVs according to MB subgroup.

All 36 WNT MBs sequenced in this study were confidently explained by mutations in at least one or more driver genes (Extended Data Fig. 6a). Somatic *CTNNB1* mutations, the hallmark feature of WNT-driven MB, were found in 86% of patients. Three *CTNNB1* wild-type MBs harboured pathogenic *APC* germline variants, explaining the WNT pathway activation seen in these patients and underscoring the need to perform genetic testing for *APC* carrier status (that is, Turcot syndrome)^25^ when WNT MB is suspected despite failure to detect mutant *CTNNB1*. Monosomy 6, a signature chromosomal alteration characteristic of patients with WNT MB, was confirmed in 83% of cases (Extended Data Figs 6a, 7a–c), demonstrating that neither *CTNNB1* mutation nor chromosome 6 loss are universally present in all patients with WNT MB. The latter has direct clinical ramifications given that positivity for either of these two features is now routinely used to clinically assign patients to this subgroup, an approach that will currently miss approximately 10–15% of bona fide patients with WNT MB.

Additional WNT subgroup-associated mutations included somatic variants that target clinically actionable *CSNK2B* (14%), *EPHA7* (8%), and subunits of the SWI/SNF nucleosome-remodelling complex (*SMARCA4*, *ARID1A* and *ARID2*; 33%; Extended Data Fig. 6a, c, d). Given the epigenetic antagonism known to exist between SWI/SNF and the PRC2 polycomb repressive complex^26^, inhibitors of PRC2 are now being evaluated for SWI/SNF-mutant paediatric cancers in clinical trials (NCT02601937) and based on our findings here, could represent a rational targeted therapy for treating WNT MB.

## SHH subgroup MB

Building on our previous work^6^, we reliably assigned at least one driver gene to more than 95% of patients with SHH MB, and revealed several insights that extend beyond genetic events that target the canonical SHH signalling pathway (Extended Data Fig. 6b). *IDH1* mutations represent a hallmark genetic event in adult patients with glioma that exhibit a distinct hypermethylation phenotype (that is, glioma CpG island methylator phenotype, G-CIMP)^27,28^. We identified six *IDH1* R132C mutations (five SHH, one WNT), consistent with a recent case report^29^(Extended Data Fig. 6e). *IDH1*-mutant SHH MBs were determined to be CIMP^+^ (Extended Data Fig. 6f, g), confirming that these mutations lead to epigenetic consequences reminiscent of those reported in other *IDH1*/*2*-mutant cancers.

Systematic Gene Ontology (GO) and pathway analysis demonstrated significant overrepresentation of somatic alterations that target histone acetyltransferase (HAT) complexes in SHH compared to other subgroups (*q* = 2.2 × 10^−3^; Extended Data Fig. 6h). Genes that encode HATs, namely *CREBBP*, *KAT6B* and *EP300*, as well as HAT complex regulatory components *BRPF1* and *KANSL1*, all exhibit recurrent, mostly SHH subgroup-restricted mutations in our series (19% of patients with SHH MB). The mechanism(s) by which deregulation of HAT activity cooperates with constitutively active SHH signalling remains poorly defined, warranting further studies to determine whether this epigenetic pathway can be exploited therapeutically.

## Group 3 and Group 4 subgroup MB

**Figure 3:**
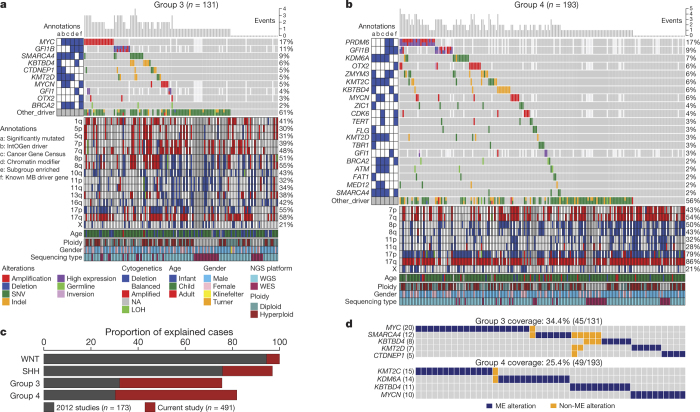
Mutational landscape of Group 3 and Group 4. **a**, **b**, Oncoprint summaries of recurrently mutated genes, structural variants, overexpression and somatic copy number variants (CNVs) in Group 3 (*n* = 131) and Group 4 (*n* = 193) MB. LOH, loss of heterozygosity; NA, not available. **c**, Bar graph depicting the proportion of cases per subgroup for which at least one driver event could be assigned. The proportion of cases explained in previous NGS studies versus the current study is shown. **d**, Mutually exclusive (ME) mutations in Group 3 and Group 4. PowerPoint slide

Re-analysis of NGS data derived from previous studies (*n* = 173)^5,9,14^indicated that less than one-third of Group 3 (32%) and Group 4 (30%) cases could be explained by at least one probable driver event (Fig. 3c), consistent with the individual estimates reported in those publications. In the considerably larger, more integrated dataset analysed here, we confidently assigned potential driver events to 76% and 82% of Group 3 and Group 4, respectively, more than doubling the proportion of explained cases per subgroup (Fig. 3a–c). As expected^11^, *MYC* amplifications were restricted to patients with Group 3 MB (17%), whereas *MYCN* amplifications were found at a comparable frequency in patients with either Group 3 (5%) or Group 4 (6%) MB (Fig. 3a, b). Similarly, structural variants leading to aberrant induction of *GFI1* or *GFI1B*^7^ were mutually exclusive and distributed in both subgroups (Fig. 3a, b). Mutual exclusivity analysis disclosed that the most prominent candidate driver events were largely non-overlapping and very few cooperating events were identified (Fig. 3d and data not shown).

Pathway analysis of recurrent genetic events revealed significant overrepresentation of genes involved in the Notch and TGFβ signalling pathways in Group 3, and in chromatin modification in Group 4 (Extended Data Fig. 5c). Aberrant Notch signalling has been repeatedly suggested in the MB literature^30,31^; however, this is, to our knowledge, the first report documenting Notch pathway mutations in samples from patients with MB. A role for deregulated TGFβ signalling in Group 3 has been suggested in our previous genomic/epigenomic studies^11,32^; however, functional studies that further substantiate these observations are still lacking.

## Epigenetic refinement of MB substructure

**Figure 4:**
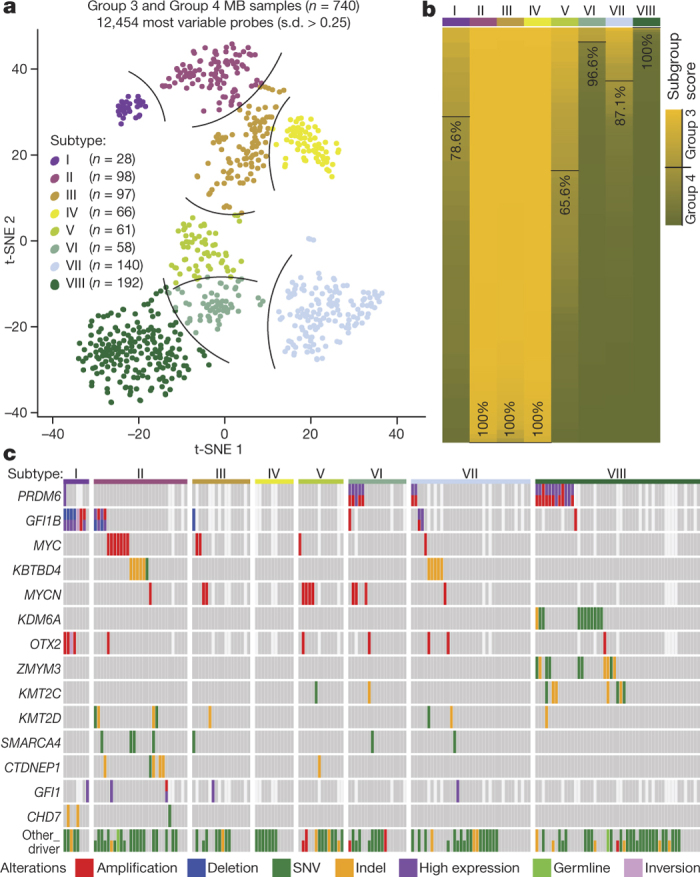
Molecular features of methylation subtypes. **a**, *t*-SNE plot depicting new methylation subtypes detected in Group 3 and Group 4 (*n* = 740 samples). **b**, Methylation subtypes proportionally summarized according to consensus Group 3 and Group 4 subgroup definitions. **c**, Oncoprint summaries of driver genetic events according to methylation subtype. PowerPoint slide

**Extended Data Figure 8:**
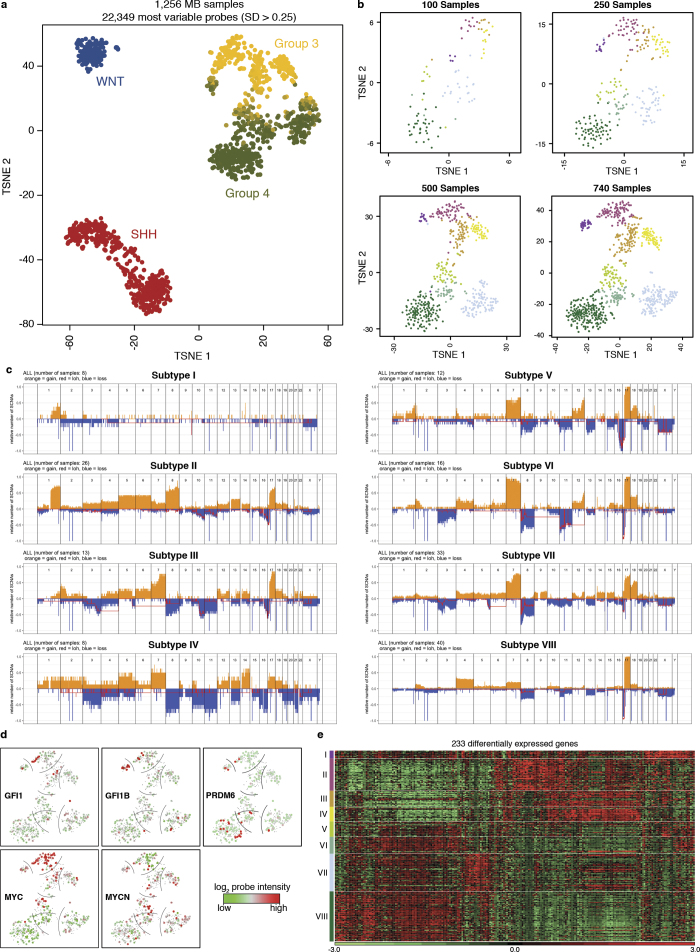
*t*-SNE analysis of Group 3 and Group 4 methylation data. **a**, *t*-SNE plot of DNA methylation array data for 1,256 analysed MBs. **b**, *t*-SNE analysis of iteratively down-sampled Group 3 and Group 4 methylation data. **c**, Genome-wide copy-number summary plots for Group 3/Group 4 methylation subtypes. **d**, *t*-SNE plots showing the relative, normalized expression intensities of *GFI1*, *GFI1B*, *MYC*, *MYCN* and *PRDM6* in methylation subtypes (*n* = 219). **e**, Expression heat map showing transcriptional diversity among new MB subtypes (*n* = 248).

The molecular composition and boundaries defining Group 3 and Group 4 MB subgroups are not as clearly demarcated as their WNT and SHH counterparts. Notable similarities between Group 3 and Group 4 have been discussed, including ambiguous cases that exhibit features characteristic of either subgroup^33,34,35^. To refine the inter- and intra-subgroup heterogeneity underlying MB subgroups, we sought to investigate molecular substructure in a series of 1,256 MBs profiled by Illumina 450 k methylation array (Extended Data Fig. 8a). Analysis of pairwise sample similarities using *t*-distributed stochastic neighbour embedding (*t*-SNE) uncovered notable heterogeneity across the cohort, especially in non-WNT subgroups. Restricting our analysis to Group 3 and Group 4 (*n* = 740) separated the parental subgroups into up to eight subtypes (Fig. 4a, b). Iterative down-sampling performed on the same dataset verified that molecular substructure seems to stabilize once 500 or more samples are included in the analysis (Extended Data Fig. 8b), exemplifying the power afforded by the high sample number included here.

Integration of methylation subtypes with sample-matched genomic and transcriptomic data revealed marked enrichment of probable driver events in specific subtypes (Fig. 4c; Extended Data Fig. 8c–e). For example, somatic events targeting the known MB drivers *GFI1B* (subtype I) and *MYC* (subtype II), as well as chromatin-modifying genes *KDM6A* and *ZMYM3* (both subtype VIII), all demonstrated remarkable subtype specificity. Broad copy-number alterations were also differentially distributed among subtypes (Extended Data Fig. 8c). Analysis of case-matched gene expression array data (*n* = 248) confirmed discriminatory transcriptional features associated with these subtypes, including aberrant expression of the MYC and GFI1 family oncogenes (Extended Data Fig. 8d, e).

## Hotspot insertions target *KBTBD4*

**Figure 5:**
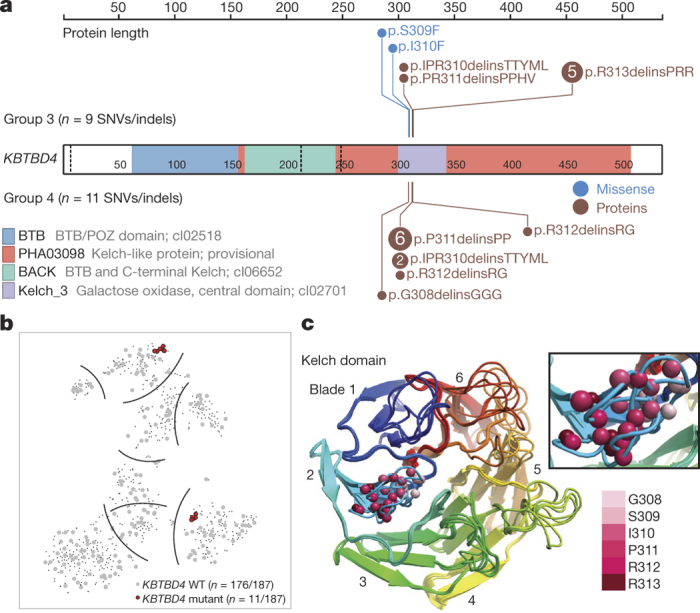
Recurrent hotspot indels target *KBTBD4*. **a**, Gene-level summary of somatic SNVs and indels targeting *KBTBD4* in MB. Mutations are independently summarized according to subgroup. **b**, Distribution of wild-type (WT) and mutant *KBTBD4* cases in methylation subtypes. **c**, Homology model of the KBTBD4 Kelch domain, highlighting the positions affected by hotspot insertions (shown as spheres). PowerPoint slide

Recurrent, in-frame insertions targeting *KBTBD4* in Group 3 and Group 4 were among the most compelling single-gene discoveries in this large dataset. Of 20 somatic *KBTBD4* variants we identified, 18 (90%) were determined to be in-frame insertions clustered across just six amino acids within the KBTBD4 Kelch domain (Fig. 5a). Notably, the predominant insertion inferred in Group 3, of proline and arginine at Arg313 (R313>PRR) differed from that observed in Group 4, an insertion of proline at Pro311 (P311>PP). Overlaying *KBTBD4* mutation status with methylation subtype assignments revealed two tightly clustered mutation groups within subtypes II (21%) and VII (14%) (Fig. 5b), ranking *KBTBD4* as the most prevalent candidate driver identified in these subtypes.

*KBTBD4* encodes a BTB–BACK–Kelch domain protein belonging to a large family of cullin-RING ubiquitin ligase adaptors that facilitate the ubiquitination of target substrates^36^. Homology modelling of the KBTBD4 Kelch domain with known structures derived from other family members (*n* = 12) verified that the MB-specific insertions observed here are unlikely to disrupt the overall structure of the Kelch domain but instead converge on the known substrate-binding interface described for other family members (Fig. 5c).

## Enhancer hijacking activates *PRDM6*

**Figure 6:**
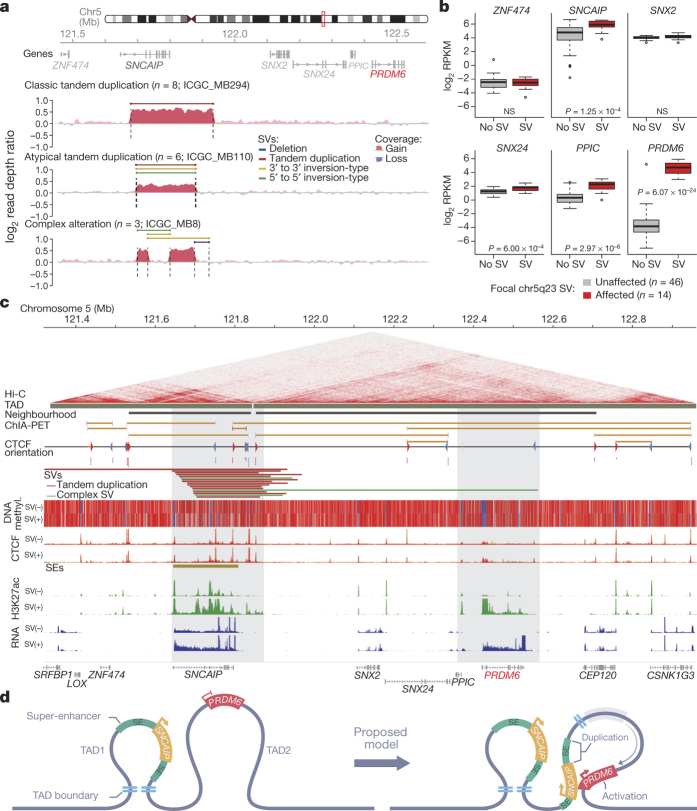
Enhancer hijacking activates PRDM6 in Group 4 MB. **a**, Summary of structural variants (SVs) targeting the *SNCAIP* locus in Group 4. **b**, Group 4 MB expression box plots of genes mapping proximal to *SNCAIP*-associated structural variants. NS, not significant. **c**, Summary of annotated chromatin interactions (Hi-C), TADs (brown bars), CTCF chromatin interaction analysis by paired-end tag sequencing (ChIA-PET) and CTCF binding orientation (red and blue arrowheads), as well as *SNCAIP*-associated structural variants, CTCF ChIP–seq peaks, Group 4-specific super-enhancers (SEs), H3K27ac ChIP–seq peaks, and RNA-seq data derived from a subset of Group 4 MBs, stratified according to underlying *SNCAIP* structural variant status. **d**, Proposed model depicting inferred molecular basis of *SNCAIP/PRDM6*-associated enhancer hijacking. PowerPoint slide

**Extended Data Figure 9:**
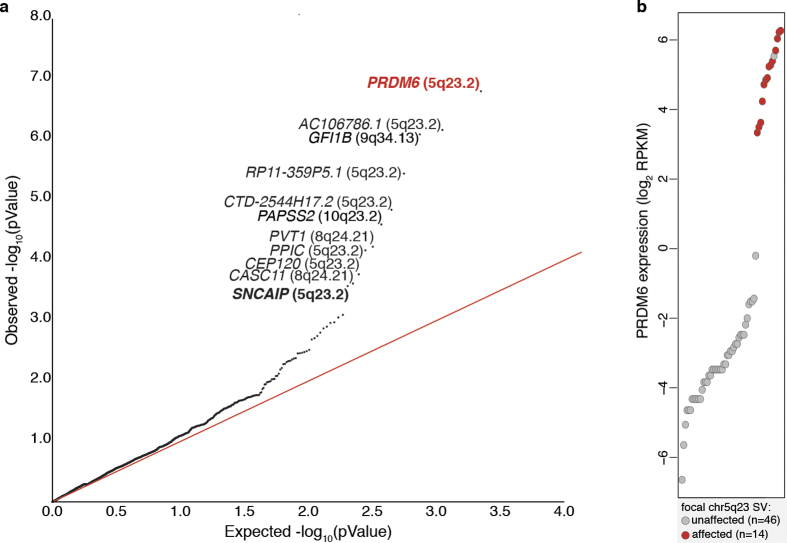
*SNCAIP*-associated enhancer hijacking in Group 4 MB. **a**, Quantile–quantile plot depicting the statistical inference of CESAM applied to systematically identify loci targeted by enhancer hijacking in Group 3 and Group 4 (*n* = 164) MB. **b**, Ascending PRDM6 expression in Group 4 MB annotated according to *SNCAIP* structural variant status.

We previously identified^7^*GFI1* and *GFI1B* as new MB oncogenes recurrently activated by ‘enhancer hijacking’ in Group 3 and Group 4. Expanding on this previous work, we recently developed *cis* expression structural alteration mapping (CESAM)^37^, an approach for systematically inferring enhancer hijacking events genome-wide by integrating gene expression and structural variant data that we interpret in the context of topologically associated domains (TADs) and enhancer annotations. Application of CESAM to MB (*n* = 164) confidently identified *GFI1B* among the most highly significant candidate genes subject to enhancer hijacking, substantiating the robustness of our method (Extended Data Fig. 9a). The top-ranking gene uncovered by CESAM, however, was *PRDM6* (chr5q23), encoding a poorly characterized SET-domain containing protein. Notably, *PRDM6* maps approximately 600 kb downstream of *SNCAIP*, a gene known to be targeted by highly recurrent, stereotypical tandem duplications uniquely restricted to patients with Group 4 MB^11^(Fig. 6a). In the context of Group 4 patients harbouring *SNCAIP*-associated structural variants analysed here, *PRDM6* expression was markedly upregulated (at least 20-fold), considerably more than any other gene mapping within the proximal TADs including *SNCAIP* (Fig. 6b; Extended Data Fig. 9b).

Using our recently published MB enhancer data^32^ and structural variant breakpoints to identify putative promoter–enhancer juxtaposition as a consequence of structural variants, we identified a significant enrichment of structural variants associated with rearrangements that link *PRDM6* to Group 4 enhancer elements (*P* < 0.0001, permutation test). The *SNCAIP* locus overlaps a strong Group 4-specific super-enhancer (Fig. 6c). Notably, the structural variants observed in *PRDM6*-activated Group 4 converge on the *SNCAIP* super-enhancer, consistent with enhancer hijacking (Fig. 6c). Integrative analysis of CTCF chromatin data revealed notable clustering of structural variant breakpoints proximal to CTCF-binding sites associated with the TAD boundary separating the *SNCAIP* and *PRDM6* loci (Fig. 6c). Collectively, these data suggest that structural variants targeting the *SNCAIP* locus disrupt the local chromatin environment to promote *de novo* interactions between the *SNCAIP* super-enhancer and gene promoters in the neighbouring TAD, thus leading to aberrant gene induction, most notably *PRDM6* (Fig. 6d).

## Discussion

Our highly integrative genomic analysis of the paediatric brain tumour MB has enabled the discovery of new cancer genes and actionable pathways, effectively assigning candidate drivers to most tumours across molecular subgroups. The sizable increase in power over previous studies has allowed us to deal more effectively with the intrinsic heterogeneity characteristic of MB, splitting the entity into molecularly distinct consensus subgroups and subtypes within them, summarizing the disease as a collection of several diseases rather than a single entity.

At the level of individual genes, novel candidate drivers were discovered in each of the consensus subgroups. Hotspot insertions that target *KBTBD4* were not featured in previous MB NGS studies, probably owing to inferior cohort sizes and insensitive indel-calling pipelines. *KBTBD4* insertions were highly specific for discrete patient subtypes that were devoid of other obvious oncogenic driver events, suggesting that these mutations are functional. Likewise, *PRDM6*—a presumed histone methyltransferase^38^ not previously implicated in MB—was identified as the probable target of *SNCAIP*-associated enhancer hijacking in Group 4, now representing the most prevalent driver alteration in this subgroup. Studies further detailing the normal, physiological cellular functions of KBTBD4 and PRDM6 and how somatic alterations targeting these genes specifically contribute to MB pathogenesis are essential and will be required to determine their potential ‘actionability’ in affected patients.

The relatively recent recognition of consensus MB subgroups has rapidly changed the way MB is studied in the research setting and how it is diagnosed and treated in the clinic^39^. Still, considerable molecular and clinical heterogeneity has been demonstrated^11,40^, suggesting that currently defined MB subgroups are likely to be an oversimplification of true molecular substructure. Methylation analysis of over 1,250 MBs discovered new tumour subtypes enriched for specific genetic and transcriptional signatures, especially those underlying Group 3 and Group 4. Definitive de-convolution of these subtypes will enable a better understanding of the developmental origins of MB, creating a path towards the efficient modelling of each individual subtype in the correct cellular context using subtype-relevant genetic perturbations. Moreover, by redefining molecular substructure as we have described here, new opportunities for improved risk-stratification tailored to treat individual patient subtypes according to their genotype are likely to emerge.

In conclusion, this study embodies an unparalleled resource of high-resolution genetic, epigenetic and transcriptional data for the childhood brain tumour MB. Our data underscore the heterogeneous, complex nature of disease subgroups and the utility of continued efforts to divulge the full spectrum of molecular mechanisms underlying MB aetiology. We anticipate that the findings reported here, combined with the future exploration and mining of this large genomics resource, will undoubtedly advance treatments and the outlook for children and families affected by this devastating malignancy.

## Methods

No statistical methods were used to predetermine sample size. The experiments were not randomized and investigators were not blinded to allocation during experiments and outcome assessment unless stated otherwise.

### Patient consent

ICGC samples: all patient material was collected after receiving informed consent according to ICGC guidelines and as approved by the institutional review board of contributing centres.

Broad and MAGIC samples: informed consent was provided by the families of patients with medulloblastoma treated at Children’s Hospital Boston (Boston, Massachusetts, USA), The Hospital for Sick Children (Toronto, Canada), and institutions contributing to the Children’s Oncology Group/Cooperative Human Tissue Network, under approval and oversight by their respective internal review boards.

St Jude samples: human tumour and matched blood samples were obtained with informed consent through an institutional review board approved protocol at St Jude Children’s Research Hospital (Memphis, Tennessee, USA).

### Bam to FASTQ and alignment

NGS data were collected from four primary sources (ICGC PedBrain^5,6,7^, PCGP^9^, MAGIC, and the Broad Institute^14^). To ensure all samples were processed with the same analysis pipelines, sequences that were not available as FASTQ files were unaligned using the SamToFASTQ tool from Picard (http://broadinstitute.github.io/picard). To avoid biases in the insert size estimation of the realignment the Bam files were name sorted before the unalignment. The subsequent alignments were done according to the standards defined for ICGC PanCancer^41^. All reads were aligned against the phase II reference of the 1000 Genomes Project including decoy sequences d5 (ftp://ftp.1000genomes.ebi.ac.uk/vol1/ftp/technical/reference/phase2_reference_assembly_sequence/hs37d5.fa.gz) using BWA-MEM (v.0.7.8 using standard values except for invoking -T 0)^42^. The raw Bam files were sorted and duplicates were marked using biobambam (v.0.0.148). Sequencing coverage was calculated using custom scripts^5^. For annotations we chose the latest compatible GENCODE version 19 (http://www.gencodegenes.org/releases/19.html).

### Variant calling

Somatic variant calling (SNVs, indels, structural variants and CNVs) was done using the DKFZ/EMBL core pipelines in accordance with ICGC PanCancer^41^. The workflow is available on the Dockstore webpage: https://dockstore.org/containers/quay.io/pancancer/pcawg-dkfz-workflow.

### SNVs

SNVs were called using the DKFZ samtools-based^42^ calling pipeline as described^3,5^ using the ICGC PanCancer version. In short, variants were first called in the tumour sample and then queried in the control sample. The raw calls were then annotated using multiple publicly available tracks such as 1000 Genome variants, single nucleotide polymorphism database (dbSNP), repeats and other elements. The functional effect of the mutations was annotated using Annovar^43^ and the variants were assessed for their confidence and split into somatic and non-somatic calls. Owing to the poor coverage of the *TERT* promoter region, variants were called with relaxed stringency manually using custom scripts.

### Indels

Raw calls for indels were obtained from Platypus (v.0.7.4)^44^. Annotation and confidence assessment was done similar to SNV processing.

### SNV and indel integration

SNVs and indels were integrated using custom scripts. Variant frequencies were calculated for the whole cohort and for each subgroup individually. To increase the already high specificity of the workflows^45^, we manually checked all functional variants (non-synonymous, stop-gain, stop-loss and splice-site SNVs and in-frame, frame-shift and splice-site indels), in genes that had at least three hits in the cohort. For manual curation, we used a custom script to take screenshots for each variant and then scored the confidence randomly at least three times for each call.

### Structural variants

Structural variants were inferred using DELLY^46^ following a standardized method across all samples (matched tumour/normal pairs) and using the cancer genome analysis workflow of ICGC PanCancer (https://dcc.icgc.org/pcawg). In brief, the same workflow was used to predict structural variants in a set of 1,105 germline samples from healthy individuals belonging to phase I of the 1000 Genomes Project (http://1000genomes.org). Predicted structural variants in the MB samples were considered somatic if they were detected in less than 1% of the 1000 Genomes Project samples. Furthermore, identified somatic structural variants were additionally required to be absent in all remaining MB germline samples from this study and absent in a set of germline samples derived from different tumour entities sequenced as part of ICGC. To exclude false positive predictions caused by low-quality mapping reads, only high-confidence calls were considered by applying additional filtering, specifically requiring at least 4 supporting sequencing read pairs with a minimum mapping quality of 20, and an structural variant size between 100 bp and 500 Mb.

### CNVs

Copy number status was estimated using ACEseq (allele-specific copy number estimation from sequencing) (K.K. *et al*., manuscript in preparation). The method uses both, a coverage ratio of tumour and control over genome windows and the B-allele frequency (BAF). It produces copy number calls as well as estimates for tumour ploidy and tumour cell content. During pre-processing of the data, allele frequencies were obtained for all SNP positions recorded in dbSNP^47^ v.135. To improve sensitivity with regards to imbalanced and balanced regions, SNP positions in the control were phased with impute2^48^. Additionally, the coverage for 10-kb windows with sufficient mapping quality and read density was recorded and subsequently corrected for GC-content and replication timing to remove coverage changes introduced by these biases. The genome was segmented using the PSCBS package in R^49^ while incorporating structural variant breakpoints defined by DELLY. Segments were clustered using k-means clustering according to their coverage ratio and BAF value and neighbouring segments that fell into the same cluster were joined. Focal segments (<9 Mb) were stitched to the more similar neighbour.

### Focal CNVs

To reliably call focal CNVs in genes of interest, we extracted overlapping breakpoints from the raw structural variant calls. To increase specificity, the events were then visualized using custom plotting scripts. Events ≤ 10 Mb were considered to be focal.

### Chromosomal aberrations

Chromosome arm-level gains, losses, and loss of heterozygosity were manually annotated using coverage plots from ACEseq that include BAF plots for loss of heterozygosity detection.

### Clonality analysis of small mutations

To assess the clonality of somatic small mutations (SNVs and indels) in high-quality WGS cases, tumour purity as well as the copy number status for somatic mutations was estimated using ACEseq (K.K. *et al*., manuscript in preparation) and the clonality was calculated (observed allele frequency/estimated purity × local copy number). A clonality of at least 1 corresponds to at least one allele being mutated after local copy number and purity correction. Since we could not confidently differentiate between clonal and subclonal events in hyperploid (*n* > 3) genomes, we restricted our analysis to samples with estimated ploidies of *n* ≤ 3.

### Expression analysis of mutated alleles

Expression of the main variants displayed in the oncoprints for samples with available RNA-seq data were determined and the frequency of the expressed allele was estimated. Specifically, variants were examined using samtools^42^ mpileup and then plotted as a fraction of the total expression observed for the respective gene. Since the representation of indels in the RNA BAM files (aligned using STAR^65^) was different than that used for DNA alignments (aligned using BWA-MEM^42^), the expression of indels was determined manually using the IGV genome browser.

### Genome-wide SNV analysis

To identify genomic regions with single recurrent mutations or clusters of recurrent mutations we used a windows-based approach in which we binned the human genome in non-overlapping windows of various sizes ranging from 50 bp to 500 bp. For each window we calculated its mutational recurrence (that is, the number of patients having at least one mutation in the given window). To estimate the background mutational rate, the ‘global’ model was used: we stratified the genome into 25 evenly sized groups of genomic windows with similar genetic and epigenetic background based on the combined vector of five genetic and epigenetic features (replication timing, gene expression level, GC content, H3K9me3 and open versus closed chromatin conformation; as described in V.A.R. *et al*., manuscript in preparation). Using these background mutational rate estimates we computed an enrichment score, binomial *P* value, and negative binomial test *P* value for each genomic window. To choose the significance cut-off that would provide reproducible results we performed cross-validations (samples were segregated by subgroup). On the basis of the results of cross-validations, we chose a combination of the window size (200 bp), test statistic and a cut-off value (binomial *P* value cut-off = 10^−25^) that ensured high precision and recall values based on precision-recall analysis. Recall was calculated as the number of regions that satisfy the cut-off in results obtained on both halves of the dataset; precision was calculated as a fraction of the recalled regions to the total number of regions satisfying the cut-off in each of the datasets. We then used the chosen parameters and executed the pipeline on the complete dataset.

### Oncoplots

The data from the variant calling workflows were summarized using custom scripts and plotted into oncoplots using the R package complex heat maps^50^. Frequencies of events were adjusted to the number of samples that could be annotated for the respective event (that is, samples where we could not call CNVs were not counted and shaded light grey for CNV relevant genes). Subgroup enrichment for specific genes was determined using Fisher’s exact test and a threshold of the Benjamini–Hochberg-adjusted *P* value (*P* ≤ 0.05).

### Significance workflows

The significance of somatic mutations (SNVs, indels) was determined using three published methods: MutSigCV^20^, IntOGen^22^ v.2.4.2 and MuSiC^21^ v.0.4. The corresponding input data formats were parsed from our custom VCF files and loaded into the respective tools. The programs were run using default settings. Significant genes were determined using the recommended significance thresholds for each of the output files.

### Copy number integration/significance

Significant copy number gains and losses (WGS samples; *n* = 352) were calculated using GISTIC^51^ v.2.0.22. We used a custom script to parse the region based output from ACEseq into a segmented data format suitable for GISTIC. Regions containing false positive recurrent events mainly around centromeres and telomeres were excluded from the analysis. The following 38 samples were excluded from the analysis owing to low data quality: ICGC_MB126, ICGC_MB143, ICGC_MB147, ICGC_MB149, ICGC_MB246, ICGC_MB256, ICGC_MB304, ICGC_MB305, ICGC_MB306, ICGC_MB62, ICGC_MB800, ICGC_MB89, ICGC_MB92, ICGC_MB94, MDT-AP-0009, MDT-AP-1200, MDT-AP-1367, MDT-AP-1369, MDT-AP-1403, MDT-AP-1405, MDT-AP-2073, MDT-AP-2110, MDT-AP-2111, MDT-AP-2115, MDT-AP-2116, MDT-AP-2307, MDT-AP-2514, MDT-AP-2532, MDT-AP-2673, MDT-AP-2719, MDT-AP-2745, MDT-AP-2774, MDT-AP-3017, MDT-AP-3019, MDT-AP-3399, MDT-AP-3402, SJMB015 and SJMB019. GISTIC was run separately for each subgroup using a length cut-off of 0.5 chromosome arms, a noise threshold of 0.3 copies, a cap of 1.5, a confidence level of 0.95 and gene GISTIC for the deletion analysis.

### Mutation signatures

Mutation signatures are calculated based on trinucleotides centred at somatic SNVs. Therefore, the immediate 3′ and 5′ nucleotides of all somatic SNVs were extracted from the reference genome and the obtained trinucleotides were converted to pyrimidine context resulting in 96 possible mutation types. Directly adjacent SNVs (multiple nucleotide variants, MNVs) were excluded for mutational signature analysis. For each sample, its mutational profile was calculated by counting the number of occurrences of each of the possible 96 mutation types. By combining mutational profiles of all samples per entity, mutational catalogues for signature extraction were compiled. The mutational profile of a tumour, and therefore, the mutational catalogue of a tumour type, is supposed to reflect a combination of mutational processes (signatures) acting on their genomes, where each mutational process has different intensities (exposures). This is modelled as a system of matrices for signatures (*P*), exposures (*E*) defining the observed mutational catalogue (*M*): *M* ≈ *P* × *E*.

To decipher ‘*de novo*’ signatures, we implemented and applied the method described previously^52^ to the mutational catalogue of each subgroup. To identify highly similar and distinct signatures, all signatures were compared across tumour types and to published signatures (available in the COSMIC database) based on their cosine similarity^16^. All detected signatures could be assigned to one of the known signatures with a cosine similarity of at least 0.85. To achieve maximum resolution per sample, we finally aimed for a sample-wise re-extraction of exposures from the mutational profiles using quadratic programming with the reference signature set as *P* and exposures in *E* as unknown variables. The resulting exposures were used for further downstream analyses and visualization. Signature probability distributions are displayed for the 96 mutation types according to the representation described previously^16^. Association of signature exposures and age at diagnosis were calculated by generalized linear models in all subgroups. Specificity of exposures for one or more subgroups (that is, significant enrichment of exposure compared to the other groups) was determined using ANOVA and post hoc Tukey’s test.

### DNA methylation array processing

DNA methylation profiling of MB samples was performed using the Infinium HumanMethylation450 BeadChip array (450 k array) according to the manufacturer’s instructions (Illumina). Data were primarily generated at the DKFZ Genomics and Proteomics Core Facility (Heidelberg, Germany) and The Hartwell Center at St Jude Children’s Research Hospital (Memphis, USA). MB subgroup status was inferred as previously described^15^ or inherited from published annotations^9,14^.

DNA methylation data of 1,256 samples presented in this study were generated from both fresh-frozen and formalin-fixed paraffin-embedded (FFPE) tissue samples. For most fresh-frozen samples, more than 500 ng of DNA was used as input material. 250 ng of DNA was used for most FFPE tissues. On-chip quality metrics of all samples were carefully controlled. Samples were also checked for unexpected genotype matches by pairwise comparison of the 65 genotyping probes on the 450 k array.

All DNA methylation analyses were performed in R v.3.3.0 (R Development Core Team, 2016). Raw signal intensities were obtained from IDAT-files using the minfi Bioconductor package^53^ v.1.18.0 using default settings. A correction for the type of material (FFPE/frozen) was performed by using the removeBatchEffect function of the limma package v.3.24.15. The log_2_-transformed intensities of the methylated and unmethylated signal were corrected individually. Beta-values were calculated from the retransformed intensities using an offset of 100 (as recommended by Illumina) and used for all downstream analyses.

The following criteria were applied to filter out probes prone to yield inaccurate methylation levels: removal of probes targeting the X and Y chromosomes (*n* = 11,551), removal of probes containing an SNP (dbSNP132Common) within five base pairs of and including the targeted CpG site (*n* = 24,536), and probes not mapping uniquely to the human reference genome (hg19) allowing for one mismatch (*n* = 9,993). In total, 438,370 probes were kept for analysis.

For unsupervised *t*-SNE analysis of 1,256 MB samples, we selected the 22,349 most variably methylated probes across the dataset (s.d. > 0.25). Pairwise sample distances were calculated by using 1 minus the weighted Pearson correlation coefficient as the distance measure. Pairwise Pearson correlation was calculated using the wtd.cors function of the weights package v.0.85. We used the probe standard deviation subtracted by 0.25 as the weight, giving more variable probes greater influence. The resulting distance matrix was used to perform the *t*-SNE analysis (Rtsne package v.0.11). The following non-default parameters were used: theta = 0, is_distance = T, pca = F, max_iter = 2000. Resulting clusters were annotated as WNT, SHH, Group 3 and Group 4 based on classification using a previously described 48 CpG signature^15^.

A similar approach was used for the unsupervised analysis restricted to Group 3 and 4 samples (*n* = 740, 12,454 most variable probes, s.d. > 0.25), and for the downsampling analysis. To ensure a similar rotation of samples, *t*-SNE analysis was performed by using the sample coordinates obtained after 150 iterations of the analysis of all MB samples as initialization points, and then performing an additional 1,850 iterations for the respective subset of samples. For the analysis of Group 3 and Group 4 samples, clusters were annotated using the DBSCAN algorithm as implemented in the dbscan package v.0.9-7. The following non-default parameters were used: minPts = 16, eps = 3.9. Subsequently, samples not assigned to any cluster were iteratively merged to their nearest cluster.

For the comparison of *IDH1*-mutated samples, we restricted the analysis to samples of the SHH subgroup that were also part of the sequencing cohort (*n* = 89). The 16,946 most variably methylated probes were used (s.d. > 0.25). One minus the Pearson correlation coefficient was used as the distance measure, and average linkage was used for hierarchical clustering.

CNV analysis from 450 k methylation array data was performed using the conumee Bioconductor package v.1.4.0. A set of 50 control samples displaying a balanced copy-number profile was used for normalization.

### Gene expression array processing

Samples for which RNA of sufficient quantity and quality was available were analysed on the Affymetrix GeneChip Human Genome U133 Plus 2.0 Array. Sample library preparation, hybridization and quality control were performed according to manufacturer’s protocols. Expression data were normalized using the MAS5.0 algorithm of the GCOS program (Affymetrix).

Gene expression differences between MB Group 3 and group 4 subtypes were analysed using available data for samples that were classified in the initial DBSCAN annotation (*n* = 219 samples). Excluding genes located on chromosomes X and Y, differentially expressed genes between subtypes were determined by the ANOVA procedure, and using Tukey’s post hoc test. Genes were considered differentially expressed if the false discovery rate (FDR)-adjusted *P* value across subtypes is <0.01, and if for at least one comparison between subtypes the absolute difference of mean expression levels was larger than 2 and *P* < 0.01 (*n* = 869 genes).

### RNA-seq and ChIP–seq data generation and analysis

RNA-seq and ChIP–seq data were generated and analysed as previously described^5,32^.

### CESAM

CESAM integrates structural variant-derived breakpoints with RNA-seq data to identify expression changes associated with breakpoints in *cis* as described in previously^37^, by performing linear regression of expression (molecular phenotype) on structural variant-derived breakpoint (somatic genotype) data. We used CESAM as described previously^37^, with some modifications. In brief, locally recurring structural variant breakpoints were assigned to bins if they fell into the same pre-annotated TAD, using TAD data from the IMR90 cell line^54^ (mean TAD size = 830 kb). A somatic genotype matrix based on ‘TAD bins’ was constructed using BEDTools (v.2.24.0)^55^ by annotating for every sample the presence/absence of breakpoints within a TAD, with ‘TAD bins’ as annotated TAD boundaries^54^. We removed genes with low expression variance (variance below the twentieth percentile). To alleviate the effect of gene dosage, we divided each the expression of each gene by the tumour/normal gene copy-number ratio (derived from ACEseq), following log_2_-transformation. We then related breakpoint presence/absence patterns with gene expression values using the FastQTL (v.2.1) algorithm^56^, by using a 2 Mb *cis*-window centred on the midpoint of the TAD. We performed 1,000 permutations with FastQTL for statistical inference^56^. To minimize the effect of confounders, we used the following covariates in the regression: (i) the total number of structural variants for each sample, to adjust for structural variant burden effects, and (ii) principal components (PC), based on principal component analysis^57^ on the somatic SCNA-derived breakpoint matrix. We used an FDR of 5% using the Benjamini–Hochberg procedure, and required more than 2-fold expression upregulation relative to controls for reporting CESAM candidate genes. Fold change was computed as the median expression in the group of structural variant ‘carriers’ compared to the median of ‘non-carrier’ control donors (median values were set to a minimum value of 1 FPKM in cases with a lower median expression level). To identify structural variants juxtaposing distal enhancers, given a set of structural variants each with two breakpoints *b*_1_ and *b*_2_—with *b*_1_ being closest to the candidate gene, overlap statistics were computed for the distant breaks *b*_2_ for the presence within 50 kb of each of a set of subgroup-specific enhancers, which was compared to an empirical background distribution by performing 10,000 randomizations of the *b*_2_ breakpoint position.

### Creating biological networks from mutation and copy-number data

To construct biologically relevant networks from mutation (SNVs, indels) and CNV data, we considered each subgroup independently and put the alterations found in each subgroup in the context of a network of known protein–protein interactions. We then used the ‘Forest’ tool from the Omics Integrator suite^58^ that solves the Prize Collecting Steiner Forest (PCSF) problem to reduce the size of the network around the genomic alterations (SNVs, indels, CNVs) used as inputs.

Assuming that disease-relevant alterations are affecting the same or closely related pathways and biological processes, considering the interaction-neighbourhood of the alterations (along with the reductionist approach of PCSF) allowed us to address two problems: (i) reduce the number of mutations to consider as functionally important for each subgroup; and (ii) help us assign rare mutations to subgroups.

### PCSF methodology

The PCSF approach seeks to find subsets of affected genes that belong to the same, possibly underappreciated or unknown, cellular processes. It begins by mapping a set of proteins of interest (here: altered genes, as they are likely to yield a gene product with altered or no function) onto a combined network of physical interactions (‘interactome’) among proteins and between proteins and metabolites derived from public databases. Each gene is associated with a ‘prize’ derived from its frequency of mutation in a subtype and each interaction is associated with a cost that is lowest for the most reliable interactions. PCSF then finds a connected subnetwork by optimizing a target function that weighs the prizes associated with input nodes (mutation/CNV) in a solution (a connected subgraph in the interactome), against ‘penalties’ associated with interactions included in the solution and aims to include as many prizes as possible. For more details about how the target function is defined, please see Tuncbag *et al*.^58^

A PCSF solution not only contains input proteins, but also interacting proteins that were not found to be altered, yet are likely to play a role in the same biological context (pathway, process, compartment, and so on) as their interaction partners (in the context of this algorithm termed ‘Steiner’ nodes”). This is particularly helpful in finding relevant pathways in the subsequent functional enrichment steps and frequently is able to link relevant molecular entities to the network that experimental methods were not able to measure. The resulting subnetwork depends on PCSF parameters, including one that effectively controls the size of the network (*β*), one that discourages highly connected *hub-nodes* in the final solution (*μ*), and one that tunes the number of individual graphs in a solution (*ω*). Each parameter combination results in a different PCSF solution. Below we describe the methods we use to choose parameters based on measures of network quality.

Aiming to build subgroup-specific networks, we used each gene that was altered (primarily SNVs, indels and CNVs) in a subgroup as input for this subgroup. Prizes were chosen based on the alteration frequency *f* of each gene within the subgroup and calculated as 1 + f. This strategy assigned similar weights to most of the genes as the alteration frequency was less than 5% within a subgroup in most cases with few exceptions (for example, *CTNNB1*, *DDX3X* and *PTCH1*).

### PCSF parameter selection

We explored and evaluated all combinations of the following parameter values for each of the subgroup-specific inputs using an interactome combining interactions from iRefIndex (v.13)^59^, HMDB^60^ (v.3.6), and the RECON^61^ (v.2) database: *β* = {1, 3, 5}, *μ* = {0.0005, 0.001, 0.005, 0.007, 0.008, 0.009, 0.01}, *ω* = {1, 2, 3}, and *D* = 7 (maximum depth searched). We discarded networks that were dominated by ‘hub-nodes’ (networks where a single node connects to more than a quarter of the nodes in the network) and selected a parameter set for each of the subgroup-network sets to perform subsequent analyses. We focused on two criteria when selecting parameter sets: (i) the number of interactions selected nodes have in the interactome (‘degree’) should be about the same for input-nodes and algorithm-added (‘predicted’) nodes, with a preference for a low average degree; (ii) we favour networks with a high input-to-predicted node ratio (number of input nodes in network divided by number of nodes added by the algorithm in network). Generally speaking, these criteria ascertain that the final networks are not dominated by high-degree nodes and that they predominantly contain proteins supported by the data. This selection process led us to choose the parameter sets {*β* = 5, *μ* = 0.007, *ω* = 1}, {*β* = 1, *μ* = 0.007, *ω* = 3}, {*β* = 5, *μ* = 0.009, *ω* = 1} and {*β* = 5, *μ* = 0.01, *ω* = 1} for networks for WNT, SHH, Group 3 and Group 4, respectively.

### Measuring robustness to parameter choices

Using these parameters, we ran the algorithm 100 times for each of the subgroups, each time slightly altering the interaction scores in the interactome to reduce the effect of the fixed scores on the selection of nodes in the network. By building the union of these runs for each subgroup, we created our final networks. Finally, for each node in each network, we calculated a robustness score as the fraction of the 100 networks that contained the node.

### Network/pathway association

To get an impression of which biological processes and pathways are overrepresented in these networks (as well as the entire mutation/CNV dataset), we performed GO enrichment (using the R package topGO (https://bioconductor.riken.jp/packages/3.2/bioc/vignettes/topGO/inst/doc/topGO.pdf) and pathway enrichment (hypergeometric tests using the Molecular Signature Database’s (MSigDb v.5.1) C2 gene sets for Canonical Pathways, Biocarta, Reactome and KEGG), followed by FDR adjustment of *P* values (using R’s p.adjust function). In the results, we focused our attention to hits with *q* < 0.01, and, to avoid very general sets, limited our final list of hits to examine pathway sets that annotate fewer than 300 genes. To link pathways to patients, we matrix multiplied the binary ‘patient × gene alteration’ data matrix with a binary ‘gene alteration × pathway’ association matrix. We used this matrix as a basis to calculate pathway–subgroup association frequencies.

### Network visualization

All network visualization was done using Cytoscape^62^(v.3). For the final display items, we reduced the size of the networks focusing on specific pathways, by filtering out nodes that were not directly associated with the pathway of interest, nodes that were not robust across Forest-runs as well as interactions (edges) with low interaction scores. While subgroup-networks were used to show subgroup-specific annotations (for example, WNT network for highlighting physically interacting SWI/SNF complex genes), we used the union of all four subgroup-networks as a base for the ‘histone lysine methylation’ network as all networks were highly enriched for this process.

### CoMEt analysis

Combinations of Mutually Exclusive Alterations (CoMEt) is a computational tool that is designed to identify mutually exclusive mutations (and other genomic events) in a binary gene by sample matrix^63^. As input, CoMEt requires three main parameters: (i) a matrix of genomic alterations (we included SNV and CNA data), (ii) *k*, the number of mutually exclusive events to be identified; and (iii) *t*, the number of groups (‘modules’) of such events. CoMEt returns roughly *t* modules containing *k* mutually exclusive genes (or slightly different numbers if the final processing step of the algorithm finds a better grouping). An important metric in analysing these modules is the coverage of the module; that is, the fraction of samples that were found to be mutated across the *k* genes. Another important detail is the number of samples in a module that are associated with alterations; this number, in the case of this particular dataset, is expected to be low owing to the low mutational frequency of most genes.

We ran CoMEt for different combinations of *k* (between 2 and 5) and *t* (between 2 and 5) on the entire dataset and subgroup-only subsets of the data. In addition, for the final analysis, we limited the algorithm to only include genes in the search that are altered in three or more samples. The main reason for setting this limit was to exclude very rare mutations, as most of these events are mutually exclusive with most other events by definition. Furthermore, when we ran CoMEt without using this limit, it was impossible to determine whether the mutual exclusivity of events in the results was a feature of the disease (or disease subtype) or a statistical artefact. To avoid this issue, we focused our analysis on modules that included genes altered in five or more samples in a particular input dataset. All modules reported by CoMEt pass a significance threshold of *P* < 1/*n* (with *n* = 100, the number of permutations we ran), meaning all of the modules presented here pass this significance threshold.

For the final display of mutual exclusivity figures, we excluded genes from the Group 3 module displayed (*k* = 4, *t* = 2) to highlight the relationship of mutually exclusive genes with higher alteration frequency; and we combined two Group 4 modules from two different CoMEt runs (*k* = 3, *t* = 2 and *k* = 3, *t* = 3) to create a larger module of mutual exclusivity (*KBTBD4* not only mutually exclusive with *KDM6A*, but also with *MYCN*, and *KMT2C*).

### Structural analysis of *KBTBD4* insertions

KBTBD4 is a member of the BTB-Kelch family proteins, which includes more than 50 members in humans^36^. All *KBTBD4* mutations observed in this study localize to the Kelch substrate-recognition domain. Although no structure has been determined for the Kelch domain of KBTBD4 (KBTBD4_Kelch_), there is an abundance of structural data about the family that allows for construction of homology models, generated with SWISS-MODEL^64^. The homology models of KBTBD4_Kelch_ adopt the six-bladed β-propeller fold with each ‘blade’ formed by a four-stranded antiparallel β-sheet. All mutations observed in our MB series occur in the loop between the second and third strands of the second Kelch ‘blade’, a known substrate recognition hotspot^36^.

### Data availability

Short-read sequencing and microarray data have been deposited at the European Genome-Phenome Archive (EGA, http://www.ebi.ac.uk/ega/) hosted by the EBI, under accession number EGAS00001001953. Genetic, epigenetic and transcriptional data can be freely explored using the PeCan (http://pecan.stjude.org/proteinpaint/study/BT.MB...Pfister%20pan-MB), R2 (https://hgserver1.amc.nl/cgi-bin/r2/main.cgi?&dscope=MB500&option=about_dscope), and PedcBio (http://pedcbioportal.org/study.do?cancer_study_id=medullo_pa_01#summary) data portals. All other data are available from the corresponding authors upon reasonable request.

## Supplementary information

Supplementary Tables This file contains a summary of the MB genomic datasets detailed in the study. (XLSX 251 kb)

Supplementary Tables This file contains a detailed summary of germline and somatic genomic alterations detected in the MB series. (XLSX 749 kb)

## PowerPoint slides

PowerPoint slide for Fig. 1
PowerPoint slide for Fig. 2
PowerPoint slide for Fig. 3
PowerPoint slide for Fig. 4
PowerPoint slide for Fig. 5
PowerPoint slide for Fig. 6
