# Supplementary figures and images for: The whole-genome landscape of medulloblastoma subtypes
Source: Nature. 2017 Jul 20;547(7663):311–7. doi: 10.1038/nature22973 (PMC5905700; doi:10.1038/nature22973)

## Slide 1
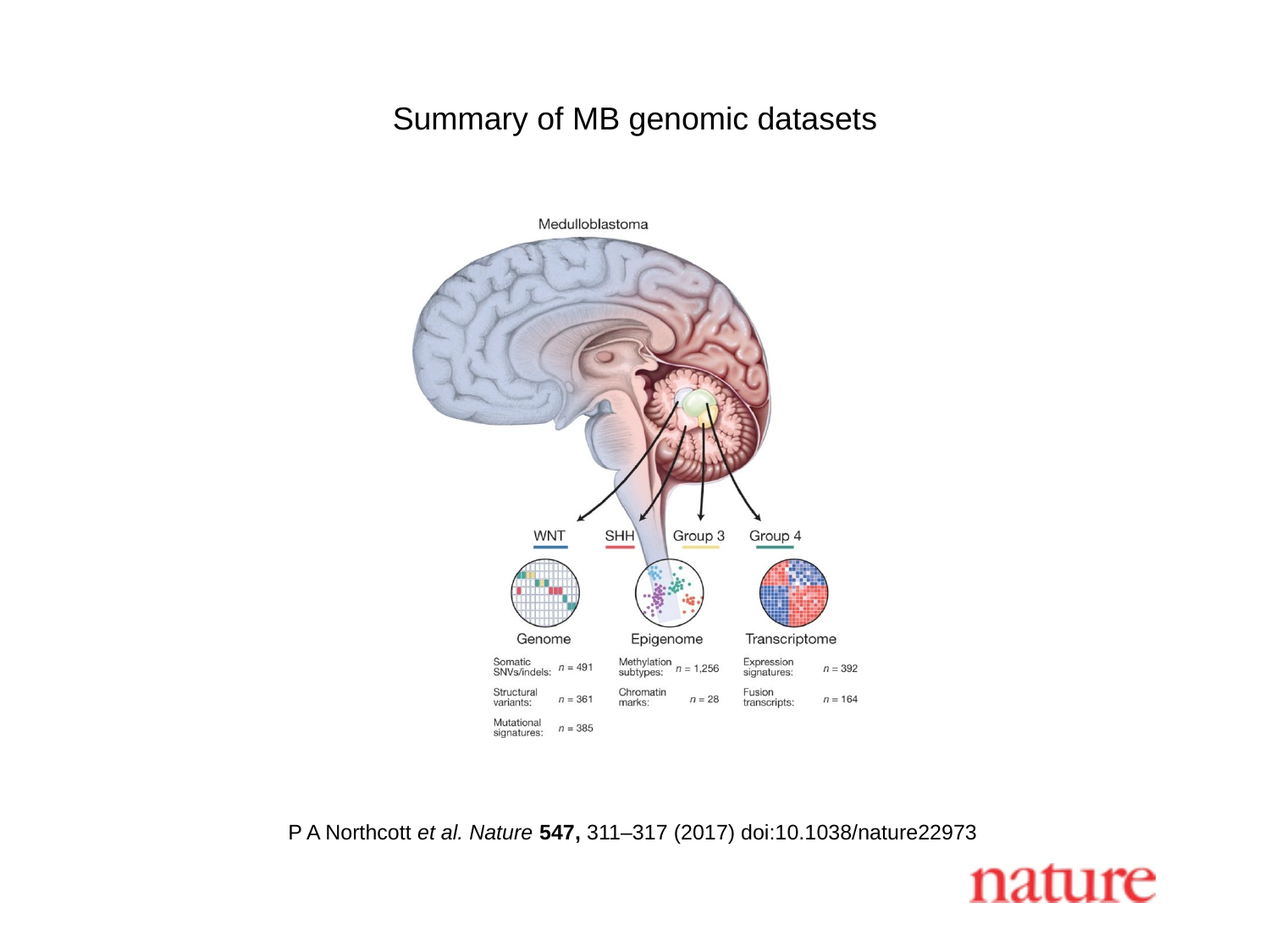

# Summary of MB genomic datasets
P A Northcott et al. Nature 547, 311–317 (2017) doi:10.1038/nature22973

Supplement: Supplementary file 3 — PowerPoint slide for Fig. 1 [file 41586_2017_BFnature22973_MOESM3_ESM.ppt]

## Slide 1
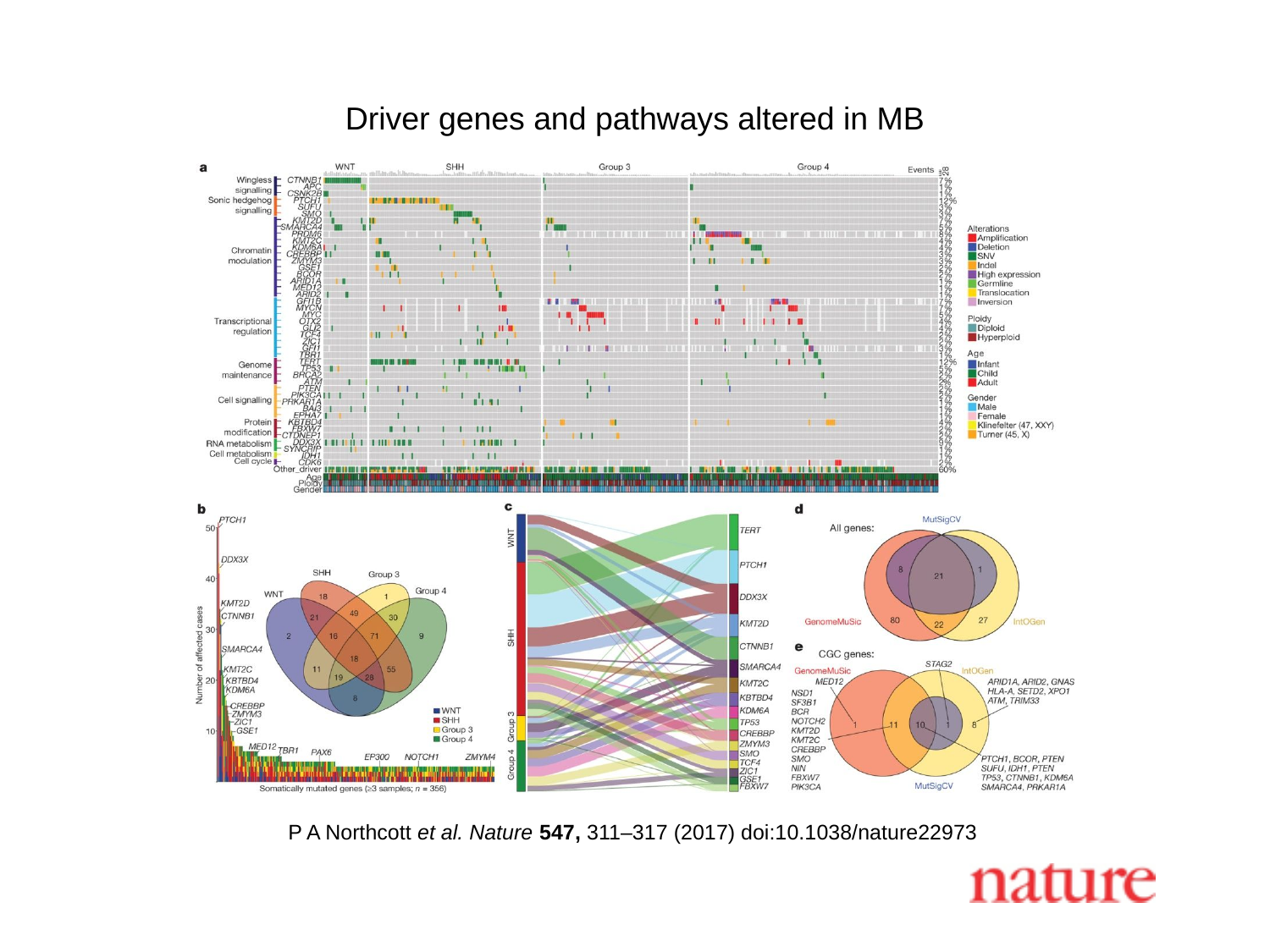

# Driver genes and pathways altered in MB
P A Northcott et al. Nature 547, 311–317 (2017) doi:10.1038/nature22973

Supplement: Supplementary file 4 — PowerPoint slide for Fig. 2 [file 41586_2017_BFnature22973_MOESM4_ESM.ppt]

## Slide 1
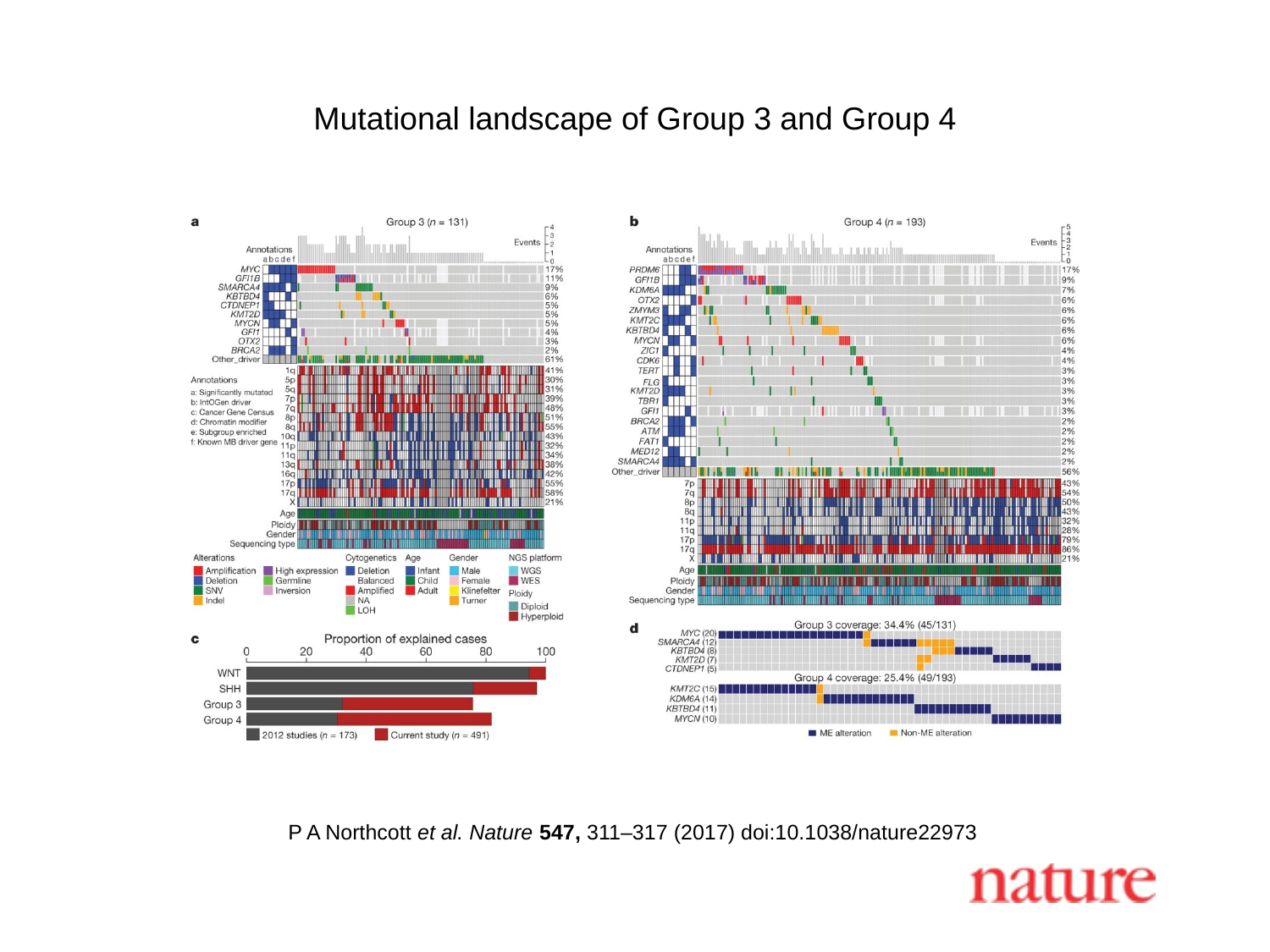

# Mutational landscape of Group 3 and Group 4
P A Northcott et al. Nature 547, 311–317 (2017) doi:10.1038/nature22973

Supplement: Supplementary file 5 — PowerPoint slide for Fig. 3 [file 41586_2017_BFnature22973_MOESM5_ESM.ppt]

## Slide 1
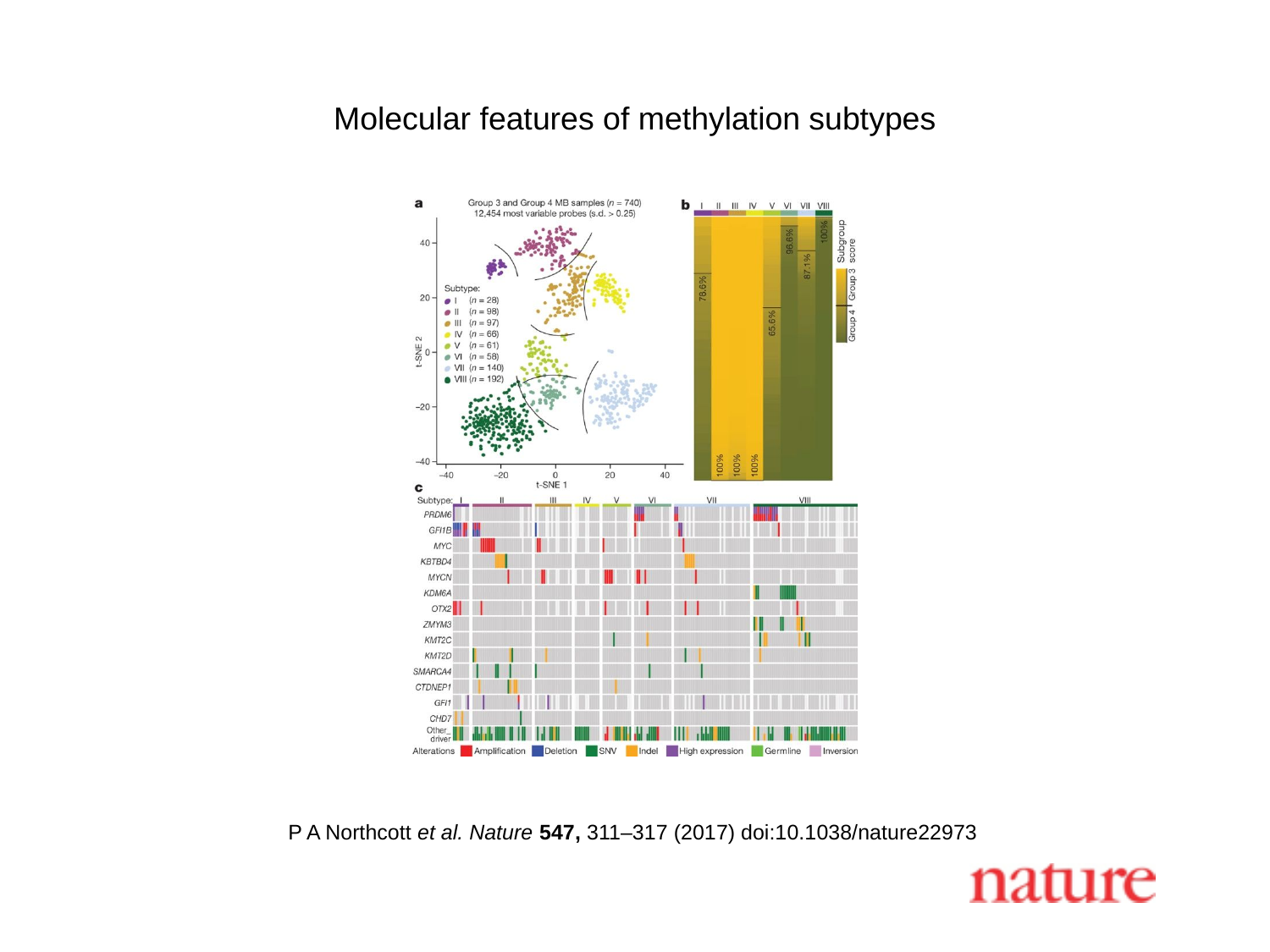

# Molecular features of methylation subtypes
P A Northcott et al. Nature 547, 311–317 (2017) doi:10.1038/nature22973

Supplement: Supplementary file 6 — PowerPoint slide for Fig. 4 [file 41586_2017_BFnature22973_MOESM6_ESM.ppt]

## Slide 1
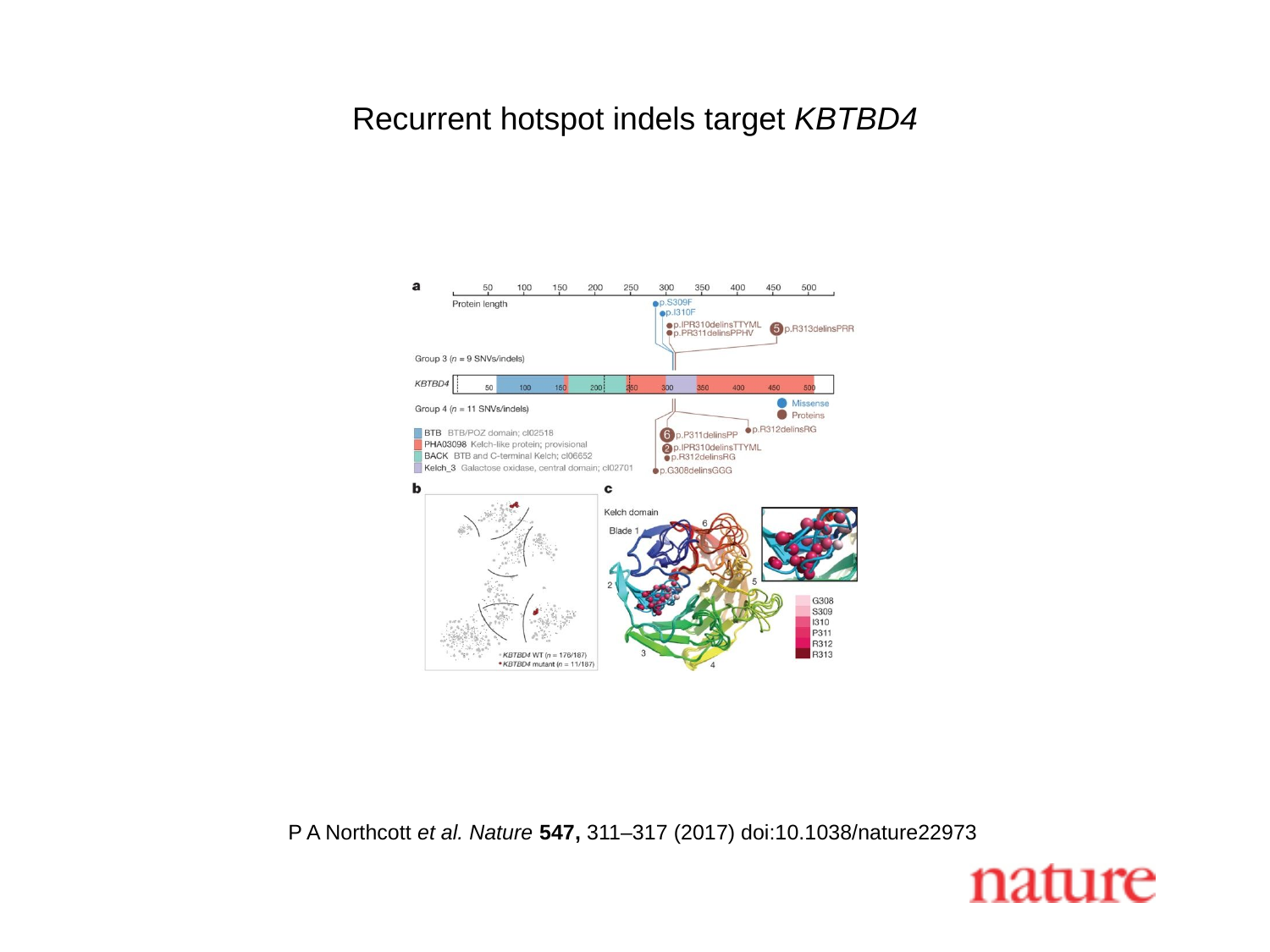

# Recurrent hotspot indels target KBTBD4
P A Northcott et al. Nature 547, 311–317 (2017) doi:10.1038/nature22973

Supplement: Supplementary file 7 — PowerPoint slide for Fig. 5 [file 41586_2017_BFnature22973_MOESM7_ESM.ppt]

## Slide 1
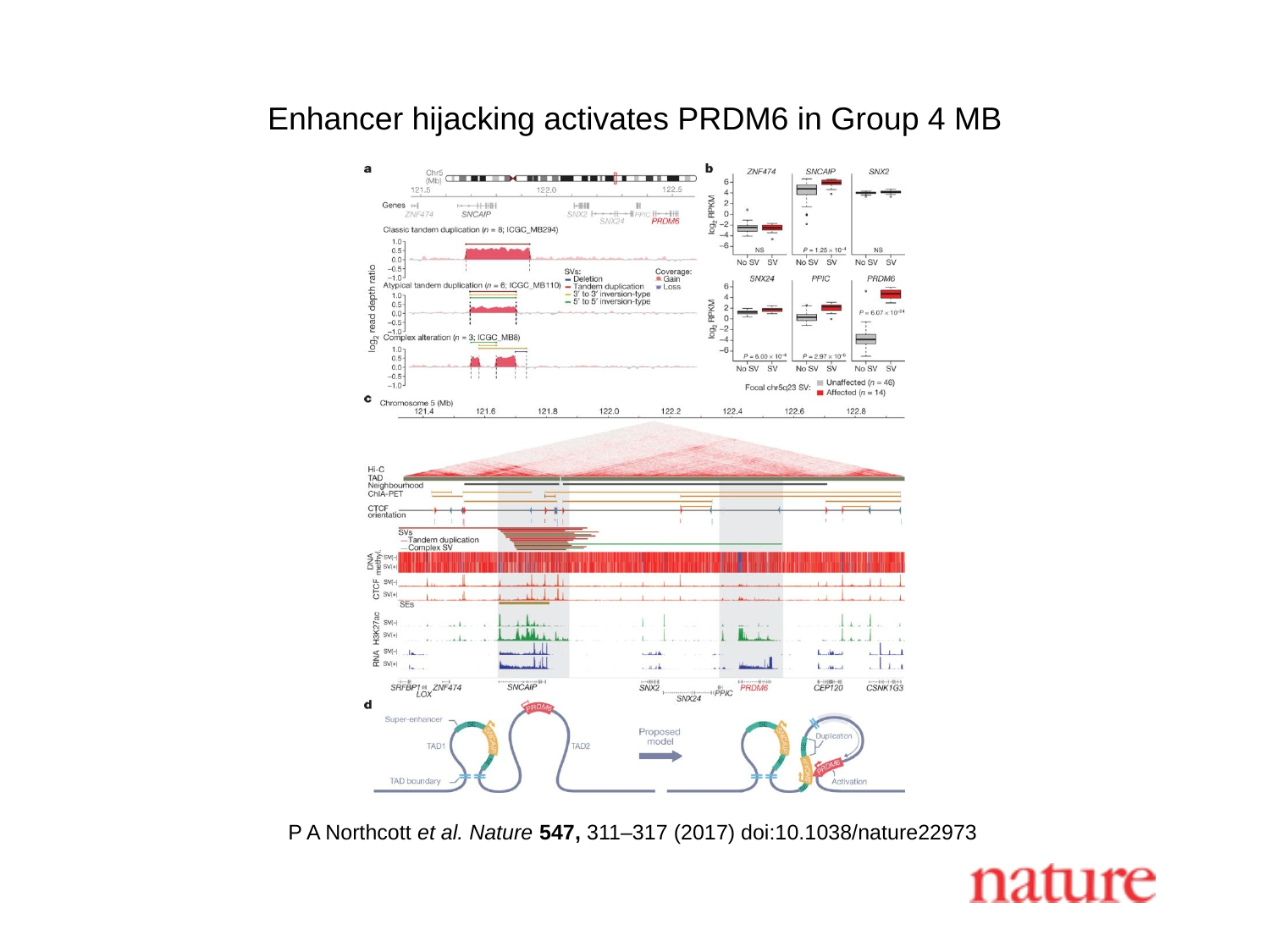

# Enhancer hijacking activates PRDM6 in Group 4 MB
P A Northcott et al. Nature 547, 311–317 (2017) doi:10.1038/nature22973

Supplement: Supplementary file 8 — PowerPoint slide for Fig. 6 [file 41586_2017_BFnature22973_MOESM8_ESM.ppt]
